# The role of BCL-2 family proteins in regulating apoptosis and cancer therapy

**DOI:** 10.3389/fonc.2022.985363

**Published:** 2022-10-12

**Authors:** Shanna Qian, Zhong Wei, Wanting Yang, Jinling Huang, Yinfeng Yang, Jinghui Wang

**Affiliations:** ^1^School of Integrated Chinese and Western Medicine, Anhui University of Chinese Medicine, Hefei, China; ^2^Gastrointestinal Surgery, Anhui Provincial Hospital, Hefei, China; ^3^School of Medical Informatics Engineering, Anhui University of Chinese Medicine, Hefei, China

**Keywords:** Bcl-2, apoptosis, cancer, autoimmunity, systematic

## Abstract

Apoptosis, as a very important biological process, is a response to developmental cues or cellular stress. Impaired apoptosis plays a central role in the development of cancer and also reduces the efficacy of traditional cytotoxic therapies. Members of the B-cell lymphoma 2 (BCL-2) protein family have pro- or anti-apoptotic activities and have been studied intensively over the past decade for their importance in regulating apoptosis, tumorigenesis, and cellular responses to anticancer therapy. Since the inflammatory response induced by apoptosis-induced cell death is very small, at present, the development of anticancer drugs targeting apoptosis has attracted more and more attention. Consequently, the focus of this review is to summarize the current research on the role of BCL-2 family proteins in regulating apoptosis and the development of drugs targeting BCL-2 anti-apoptotic proteins. Additionally, the mechanism of BCL-2 family proteins in regulating apoptosis was also explored. All the findings indicate the potential of BCL-2 family proteins in the therapy of cancer.

## 1 Introductions

Apoptosis is a genetically regulated form of cell death, which is responsible for the programmed culling of cells during the process of maintaining normal development and homeostasis in eukaryotes (1). As an important physiological process, apoptosis selectively clears cells and is widely considered to be a crucial mechanism for regulating death. It occurs not only when cells are damaged or under external stress but also during normal cell development and morphogenesis (2). So far, researchers have identified two major apoptotic pathways, i.e., the exogenous and endogenous pathways, both of which have cell death as their ultimate goal (3). Among them, the exogenous pathway, also called the “death receptor” pathway, is activated by pro-apoptotic stimuli outside the cell, whereas the endogenous (mitochondrial) pathway, as the name suggests, is activated by the intrinsic mechanisms of the cell itself (3). Evasion of apoptosis can lead to the development of cancer, which is fundamental to cancer pathogenesis (3).

Members of the B-cell lymphoma 2 (BCL-2) protein family are key regulators with pro- and anti-apoptotic activities. These regulators are held in a fine, delicate balance in healthy cells. Actually, they can cause cells to irreversibly head toward cell death or, conversely, allow cells to permanently escape apoptosis and make themselves a malignant clone (4). Over the past two decades, members of the BCL-2 family have been identified and classified according to their domains and functions. Characterized by the presence of short conserved sequence regions (BCL-2 homology [BH] motifs), the proteins of BCL-2 family are classified into three subgroups, i.e., the anti-apoptotic/pro-survival proteins represented by BCL-2 and BCL-XL, the pro-apoptotic proteins represented by BAX and Bak, and the pro-apoptotic BH3-only proteins represented by BAD and BID (5).

Generally, malignant tumors are mainly treated by hand surgery, radiotherapy, chemotherapy, immunotherapy, and targeted therapy. Of these, chemotherapy is currently one of the most effective, despite its many side effects (2), and small-molecule inhibitors are a representative strategy for cancer-targeted therapy. The mechanism of action of the targeted therapy is that drug molecules cause loss of protein function by occupying the binding pocket or active site of the target protein (4). Since the expression of Bcl-2 protein in tumor cells is much higher than that in normal cells, inhibitors targeting it have little effect on normal cells. Consequently, overcoming the resistance of tumor cells to apoptosis by inhibiting the BCL-2 anti-apoptotic protein is a novel therapeutic regimen based on tumor pathogenesis (6). Due to their multiple functions in cancer, BCL-2 family proteins have become interesting targets for anticancer drugs, which can protect tumor cells from apoptosis under various endogenous and exogenous pressures (7). For instance, Venetoclax (ABT-199), the first commercially available selective BCL-2 inhibitor, is primarily approved for treating chronic lymphocytic leukemia (CLL) and acute myeloid leukemia (AML) (8, 9). Targeting anti-apoptotic proteins of the BCL-2 family can promote apoptosis to overcome tumor chemotherapy resistance (10–12).

However, mutations in drug binding sites are a common mechanism by which malignant cells evade therapy. Studies have shown that mutations in the BCL-2 and BAX proteins are frequently detected in several types of cancers, suggesting that they play crucial roles in elucidating molecular mechanisms driving oncogenic transformation (13) and drug resistance (14). For example, the BCL-2 F104L and F104C mutations were observed as venetoclax-resistance mutations in a mouse tumor model (15) and induced drug tolerance in human cell lines (16). Compared to wild-type BCL-2, this mutation reduces the binding affinity of BCL-2 to venetoclax without altering the affinity for BAX and BIM, thus allowing the mutant protein to maintain the pro-survival effect (17). Therefore, mutations in BCL-2 family proteins require further studies to address the role of BCL-2 mutations in disease etiology, their pathways to pathogenesis, and the impact of these mutations on drug response.

Presently, the purpose of this review is to highlight the current findings on the role of BCL-2 family proteins in regulating apoptosis and the development of drugs targeting BCL-2 anti-apoptotic proteins. Additionally, the mechanism of BCL-2 family proteins in regulating apoptosis was also discussed. All the findings indicate the potential of BCL-2 family proteins in the therapy of cancer with the aim of improving patient survival.

## 2 The structural domains of BCL-2 family proteins

Generally, the BCL-2 family proteins possess four conserved BCL-2 homology (BH) domains, named BH1, BH2, BH3, and BH4, which are made up of eight α-helical fragments linked together (5). The highly conserved BH domain is an important basis for the function of BCL-2 family molecules. According to the homology and function of each protein, the BCL-2 family of proteins found in mammals is divided into three subfamilies, i.e., anti-apoptotic proteins represented by BCL-2 and BCL-XL, pro-apoptotic proteins represented by BAX and BAK, and BH3-domain-only proteins represented by BAD and BID (18).

Among them, anti-apoptotic proteins exert anti-apoptotic activity and share a sequence homology particularly within four regions, BH1 (BCL-2 homology)–BH4 (19). Pro-apoptotic proteins exert pro-apoptotic activities and share sequence homology at BH1, BH2, and BH3, but not at BH4, although significant homology at BH4 is also noticed in some members. In addition, BH3-domain-only proteins have pro-apoptotic activities and share a sequence homology only within BH3 and are thus called BH3-only proteins (20).

Moreover, during these domains, the BH4 domain is responsible for stable binding and covers the BH3 domain, thus inhibiting the pro-apoptotic effect of BCL-2 family proteins. Also, the BH3 domain is a necessary structure for the combination of pro-apoptotic proteins and anti-apoptotic proteins to form dimers and is also a necessary domain for the pro-apoptotic function of BCL-2 family members. With respect to the BH4 domain, it plays a significant role in the anti-apoptotic function of BCL-2 family proteins since once the BH4 domain was knocked out, BCL-2 completely lost its anti-apoptotic ability and had no effect on BCL-2 binding to form a dimer. The BH4 domain is also unique to apoptotic proteins. Its deletion can cause the protein to lose its anti-apoptotic function and even produce a pro-apoptotic mutant. In addition, the BH4 domain interacts with other effector molecules and apoptosis regulators outside the BCL-2 family to participate in angiogenesis, autophagy, and other apoptosis pathways (21). **Table 1** summarizes the subfamily groups, domains, and relative molecular weights of the BCL-2 family proteins.

**Table 1:** Subfamily groups, domains, and relative molecular weights of the BCL-2 family proteins.

Subfamily group	Protein name	Structural domain	Molecular weight
Anti-apoptotic proteins	BCL-2 BCL-XL MCL-1 BCL-W BFL-1	BH1.2.3.4 BH1.2.3.4 BH1.2.3 BH1.2.3.4 BH1.3	26 kDa 30 kDa 37 kDa 18 kDa 21 kDa
Pro-apoptotic proteins	BAX BOK BAK BCL-XS	BH1.2.3 BH1.2.3 BH1.2.3 BH3.4	21 kDa 25 kDa 23 kDa 19 kDa
BH3-domain-only proteins	BAD BIM PUMA BID	BH3 BH3 BH3 BH3	24 kDa 25 kDa 26 kDa 22 kDa

### 2.1 Anti-apoptotic proteins of the BCL-2 family

Traditionally, the anti-apoptotic proteins of the BCL-2 family are determined by their anti-apoptotic activities and the presence of BH4 and transmembrane domains for anchoring to cellular membranes (22). Through affecting mitochondrial membrane permeability change, the anti-apoptotic proteins of the BCL-2 family participate in the regulation of apoptosis. Herein, we focus on the BCL-2 anti-apoptotic subfamily and known isoforms. These anti-apoptotic multidomain proteins contain BCL-2, BCL-2-like 1 (BCL-XL), BCL-2-like 2 (BCL-W), BCL-2-related protein A1 (BFL-1), and myeloid cell leukemia-1 (MCL-1) mainly present in mitochondria (23–25).

#### 2.1.1 BCL-2

BCL-2 is the most characteristic anti-apoptotic protein in the BCL-2 protein family. The protein size is 26 ku and is located on chromosome 18. It can inhibit apoptosis by forming a heterodimer with BAX and ensure cell survival by regulating the Ca^2t^ concentration and antioxidant effect (26). Additionally, it can also inhibit the activities of caspase-9, 3, 6, and 7 (27), thereby inhibiting apoptosis, prolonging the survival time of tumor cells and causing malignant transformation of cells (28).

#### 2.1.2 BCL-XL

The BCL-XL gene, which has a similar structure to the BCL-2 gene, was first cloned in 1993 because the chicken gene could be crossbred with the mouse BCL-2 cDNA gene (29). The BCL-XL protein is the first protein whose spatial structure has been elucidated in the BCL-2 protein family (29). Among multiple BH domain proteins, BCL-XL has the longest sequence in the region spanning the BH domain (30). It is a protein that locates in the outer membrane of mitochondria and nuclear membrane transmembrane protein that binds to nuclear proteins and regulate transcription factor activity (31). The high expression of BCL-XL is combined with the proliferation, growth, and metastasis of tumor cell invasion, tumor stem cell phenotypic maintenance, angiogenesis, and invasive increase (32), which is closely related to apoptosis resistance. Additionally, the expression levels of BCL-XL protein is higher in cancer cells than that in the standard cells. Through reducing the release of mitochondrial cytochrome C, the BCL-XL protein prolongs the survival time of transplanted cardiac myocytes (33).

#### 2.1.3 BCL-W

First discovered by Gibson et al. in 1996 (34), BCL-W is found on human chromosome 14q11 and is highly conserved between humans and mice. It has a similar sequence with BCL-XL (35) and has higher conformational flexibility. BCL-W interacts with BAX and BAK as well as several BH3-only proteins like BAD (36), BIM (37), and PUMA (38), as shown by co-immunoprecipitation. Since the protein BCL-W is mainly located in mitochondria and exists in the form of peripheral membrane protein, BCL-W binds to the mitochondrial outer membrane in normal corpuscles and is inserted into the mitochondrial membrane structure in the course of apoptosis (39). The level of BCL-W is controlled by a variety of signaling pathways, and the transcriptional regulatory library is largely dependent on the cell and developmental environment (40). In addition, the half-life of BCL-W is short. As a highly regulated protein, BCL-W helps aging and drug-resistant cells survive (41). Its non-apoptotic role in promoting cell migration and invasion is also elucidated (42).

#### 2.1.4 BFL-1

BFL-1 is also known as GRS or BCL2A1 (murine) and encodes 175 amino acids with a relative molecular mass of 20,100, which is among the smallest molecules in the BCL-2 family (43). BFL-1 includes highly conserved BH1 and BH2 domains and conserved BH3 and BH4 and can bond to proapoptotic proteins like NOXA and BID (44). It locates in mitochondria and performs anti-apoptotic functions (45). In addition, the proliferation of macrophages and mast cells in the allergic reaction (46) was promoted by BFL-1 binding to the Beclin-1 protein. Depolarization of the mitochondrial membrane and release of apoptotic factors were also prevented by this mechanism by inhibiting BAX and BAK dimerization by the anti-apoptotic member BFL-1 (43). In this way, the downstream caspase pathway is blocked by BFL-1, and apoptosis is inhibited (47). The tumor necrosis factor (TNF) can cause bleeding, necrosis, and killing of tumor tissue, which can cause anti-infection inflammatory response and take a role in regulation and induction of immune cells. Normal cells are not affected, and BFL-1 may be involved in TNF-A resistance in normal cells.

### 2.2 Pro-apoptotic proteins of the BCL-2 family

As a rule, proapoptotic multidomain proteins include BAK, BOK, BAX, and BCL-XS and contain feature regions of BCL-2 BH domains, namely, BH1~4. Apoptosis is mainly related to MOMP. If MOMP occurs, pro-apoptotic proteins located in the mitochondrial membrane gap are released into the cytoplasm, triggering a caspase cascade that promotes cell apoptosis.

#### 2.2.1 BAX

BAX is the first BCL-2-associated protein identified by immunoprecipitation and yeast two-hybrid screening (48). BAX proteins mainly have domains with familial structural characteristics: BH1, BH2, and BH3 (49). The activated BAX can form pores in the outer membrane of mitochondria through oligomerization, which is MOMP (50). BAX and BCL-2 can form isodimers separately or interact with each other to form isodimers. The level of their proteins is directly related to the regulation of apoptosis: when BAX increases, cell apoptosis will be promoted. Increased BCL-2 inhibited apoptosis (51). BAX usually occurs in cytoplasm. BAX binds to the mitochondrial membrane, forming a permeable membrane and establishing a mitochondrial membrane channel (52). Apoptosis is regulated by inhibiting the release of cytochrome C by inhibiting BAX insertion into the mitochondrial membrane or directly or indirectly inhibiting the activity of BAX channels. When cells respond to apoptosis signals such as injury or stimulation, BAX relocates on the surface of mitochondria and plays a role by disrupting the integrity of the mitochondrial membrane (53). The activity of BAX is mainly inhibited by tumor P53 and other members of the BCL-2 family in the cytoplasm regulation of accumulation amount (54).

#### 2.2.2 BAK

BAK is another major apoptotic effector which is the transmitochondrial membrane protein, activated by apoptotic signals (55). BAK has high homology with BH1, BH2, and BH3 of BCL-2 (53). Therefore, BAK plays an important role in apoptosis and is an important regulatory factor in the apoptosis process (56). BAK protein encoded by BAK gene can promote apoptosis directly or indirectly: 1) BAK alone can promote apoptosis by inhibiting the apoptosis activity of BCL-2 and BCL-XL (57), or BAK neutralizes the apoptosis inhibitory protein of virus, promoting apoptosis (58). 2) BAK either directly activates the apoptosis pathway (59) or is activated as part of the cell death process to promote apoptosis. It is activated by exposure to its internal BH3 domain and quickly sends bio-oligomerization then disrupts the stability of the mitochondrial outer membrane (60). BCL-2 anti-apoptotic member proteins could not inhibit protein activity, and mitochondrial membranes could trigger apoptosis independently of BAK and BAK osmosis and downstream (61). Previous research findings showed that the lack of BAK expression is related with gastric cancer (62), skin cancer (63), pancreatic cancer (64), and colorectal cancer (65).

#### 2.2.3 BOK

BCL2-associated ovarian killer (BOK) protein was first identified by screening rat ovarian fusion cDNA libraries using yeast 2 hybridization with anti-apoptotic MCL-1 (66) as bait. Is a highly conserved member of the BCL-2 family, maintaining the same sequence and structure as members of the multi-domain BCL-2 family (67). In normal cells, it is primarily located in the endoplasmic reticulum. BOK is not as stable as BAX and BAK, and its cellular level is regulated by the endoplasmic reticulum-associated degradation (ERAD) pathway (68). Human BOK is the only protein with a leucine sequence in BCL-2, and high expression of BOK accelerates morphological changes in mitochondria (69), ER (70), and Golgi bodies (67). This finding suggests that BOK may play an important role in shaping organelle membranes and suggests the presence of nuclear output signals in the BH3 domain.

#### 2.2.4 BCL-XS

BCL-XS is a small fragment mRNA product (30) of BCL-X, a pro-apoptotic fellow of the BCL-2 family, and a reverse regulator of BCL-2 and BCL-XL (26). It is present in mitochondria, including BH3, BH4, and transmembrane regions, and is induced by apoptosis and caspase activation in a BH3-dependent manner through the liberation of cytochrome C. It contains the BH3 and BH4 domains and a transmembrane region and localized in the mitochondria and induces apoptosis in a BH3-dependent manner and caspase activation (71) by a mechanism involving cytochrome c release (72). Lindenboim et al. found (73) that BAX could induce apoptosis alone and trigger apoptosis mechanism by using embryonic fibroblasts from mice lacking apoptotic members and apoptotic bodies in multiple domains of BCL-2, and the apoptotic mechanism induced by BCL-XS depended on the activation of BAK.

### 2.3 BH3-domain-only proteins

BH3-only protein is the most important way for cells to respond to external apoptotic signals including BAD, BIM, BID, and PUMA which receive apoptotic signals, the expression of the BH3-only protein increases, and posttranslational modification occurs (74), which promotes apoptosis through two mechanisms: one antagonizes anti-apoptotic proteins in the BCL-2 family and the other activates pro-apoptotic proteins BAX and BAK (75). Proteins containing only BH3 domains can induce BAX and BAK to be active and inhibit BAX and BAK transmission through mitochondria by activating and neutralizing their survival proteins (76).

#### 2.3.1 BAD

BAD and BCL-2 remain with the BCL-2 gene, and their role is to promote apoptosis. The pro-apoptotic effect of the BAD gene is realized by the formation of heterodimers between the expression products of BAD gene and BCL-2 gene with the inhibition of the anti-apoptotic effect of BCL-2 (77). BAD gene plays a regulatory role in promoting apoptosis mainly through phosphorylation of Ser112, Ser136, and Ser155 (78). Protein kinases downstream of the AKT signaling pathway are activated. Regulated by the transfer chain, phosphorylated BAD forms a dimer that cannot function in the mitochondria (79). High expression of BAD is closely related to tumor cell apoptosis. Therefore, promoting BAD expression has attracted more and more attention in tumor treatment.

#### 2.3.2 BIM

BIM usually exists in an inactive state with microtubules or complexes with other pro-survival proteins (80). Also, it exists in epithelial cells, reproductive cells, hematopoietic cells, nerve cells, and other normal tissue cells (81) and plays a crucial role in the occurrence of tumors and the prevention of autoimmunity. The protein of BIM can promote apoptosis only when it dissociates from the cytoplasmic protein complex after being stimulated (82). Additionally, BIM can promote cell DNA damage (83) and play an important role in transmitting death signals (84). The upregulation of BIM gene expression can more effectively increase the apoptosis rate of tumor cells.

#### 2.3.3 BID

BID is a pro-apoptotic protein whose typical biological action is to promote apoptosis. Full-length BID is generally inactive under normal physiological conditions (85). The BID activation pathway is generally activated by caspase 8 in response to FAS/TNF-R1 death receptor signaling (86). After activation, BID exists in mitochondria, which can accelerate the release of cytochrome C (87) and further activate downstream caspases. The BH3 domain of BID works in conjunction with BCL-2, BCL-XL, and BAX to attenuate cell survival induced by BCL-2 and BCL-XL (88). Normally, intact BID is present in the cytoplasm, but as cells begin to divide, BID is cut open and transported to the mitochondria. The BID protein-induced mitochondrial cytochrome C release was independent of mitochondrial permeability alteration channels and did not cause mitochondrial swelling. The BID protein usually works in conjunction with BAX (89) protein to accelerate the fusion of BAX with mitochondria, thereby altering the structure of BAX and enhancing the mitochondrial damage induced by BAX (90).

#### 2.3.4 PUMA

PUMA, a pro-apoptotic gene discovered in colon cancer cells in 2001 (91), also called BBC3 (BCL-2 binding component 3) (92), locates in the outer membrane of mitochondria. PUMA interacts with BH1, BH2, and BH3 of BCL-2/BCL-XL (93) on the mitochondrial membrane to promote apoptosis, thereby removing the inhibitory effect of the BCL-2 anti-apoptotic protein subfamily on other BCL-2 family proteins. It can also act directly on mitochondria together with BAX/BAK and promote cell apoptosis by acting directly on BAX/BAK (94). Autophagy cell death is another considerable biological mode of cell death, which is an important process of turnover of intracellular material in eukaryotes (95). Studies have shown that PUMA (96) can participate in mitochondrial autophagy by the binding function of the BH3 domain (70) and PUMA can induce mitochondrial autophagy by selective removal of mitochondria by BAX/BAK (97). PUMA induces both mitochondrial autophagy and apoptosis, so selective mitochondrial autophagy can enhance apoptosis.

**Figure 1:**
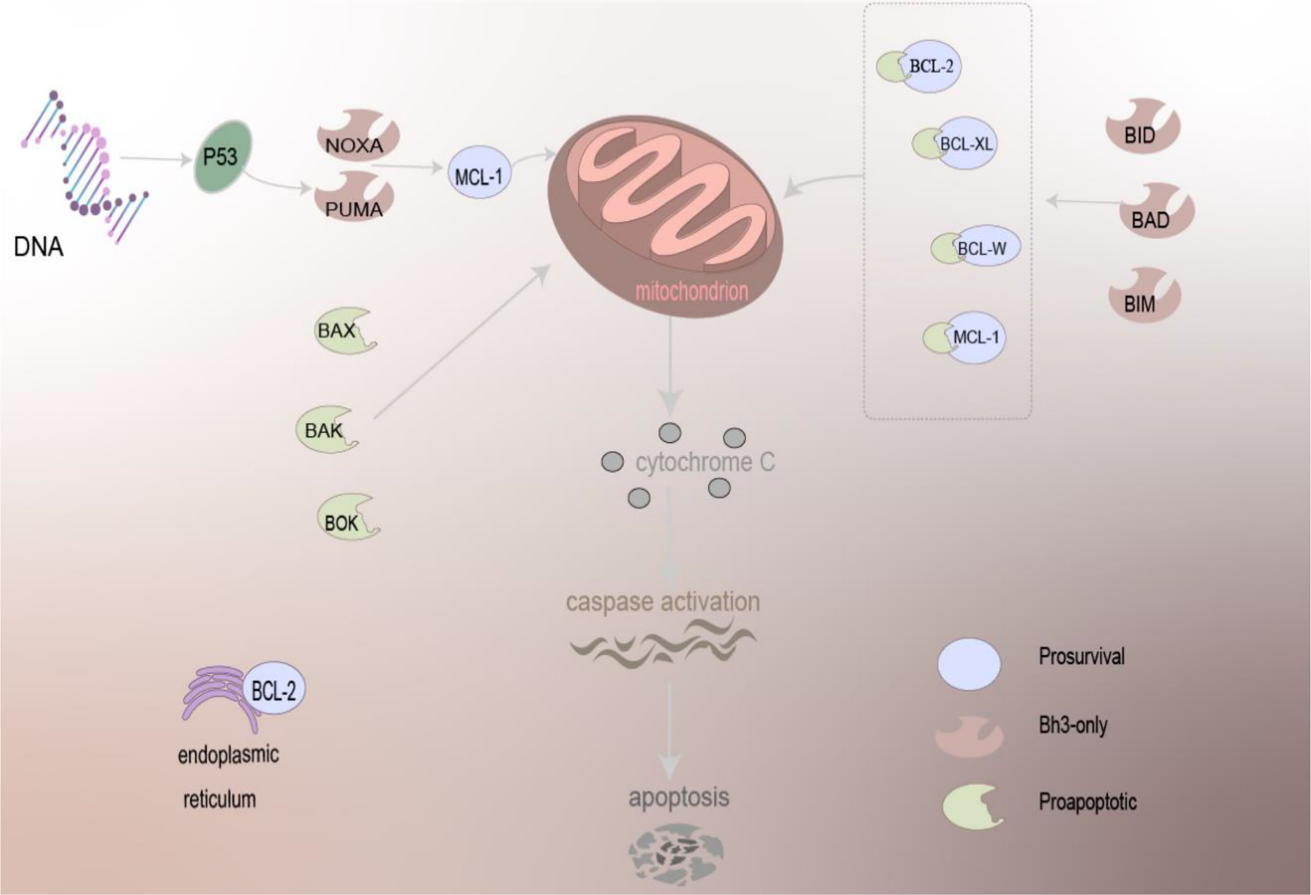
The mechanism of BCL-2 family proteins in regulating apoptosis. Driven by BCL-2 family proteins, which are primarily localized to mitochondria and present on the ER, pro-apoptotic family proteins can act directly on the mitochondria to trigger the apoptosis mechanism, and anti-apoptotic proteins need to work together with other proteins to cause apoptosis, release cytochrome C, and activation of caspase of apoptosis.

To sum up, BCL-2 can localize to the mitochondria, endoplasmic reticulum, and nuclear membrane. The apoptosis involved in the regulation is very complex, and there are many molecules involved. The mechanism of the BCL-2 family-regulated apoptosis is depicted in **Figure 1**.

## 3. The role of the BCL-2 family proteins in regulating apoptosis of cancers

**Figure 2:**
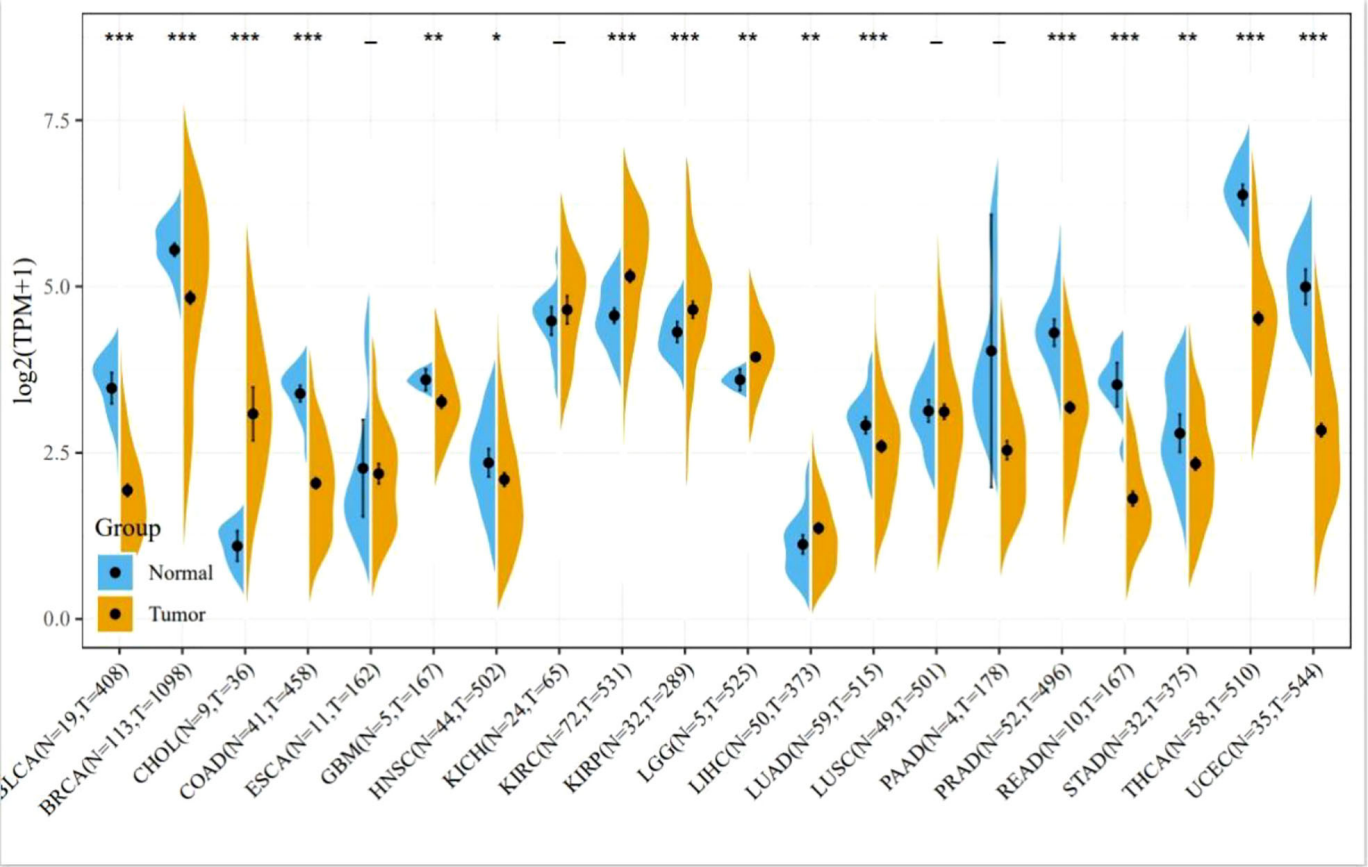
Expression of BCL-2 in various tumors. Comparisons of BCL-2 expression levels between tumor tissues from TCGA database and normal tissues from the GTEx database (*P < 0.05, **P < 0.01, ***P < 0.001).

Apoptosis inhibition is the main reason for cancer proliferation, and BCL-2 family members play a central role in regulating apoptosis. Oncogenesis of cancers is usually associated with an abnormal expression of members of the BCL-2 family proteins. **Figure 2** summarizes the expression of BCL-2 protein in various cancers including breast cancer, gastric cancer, prostate cancer, and hepatocellular carcinoma. Obviously, the expression level of BCL-2 protein in many cancer cells significantly increased. Additionally, high anti-apoptotic protein also breaks the mechanism of normal cell apoptosis, making tumor cells insensitive to apoptotic signals and gain growth advantages and overgrow.

### 3.1 BCL-2 and gastric cancer

Gastric cancer is a malignant tumor originating from gastric mucosa. Ishida et al. (98) for the first time used deoxynucleotide terminal transferase-mediated DUtP-biotin gap terminal labeling technology and found that apoptosis exists in the tissues of patients with gastric cancer. They found that apoptosis has a very important relationship with the development of gastric cancer, and many cells in precancerous lesions undergo apoptosis. Actually, during the occurrence of gastric cancer, the apoptotic effect of the general gastric mucosa will be greatly weakened, resulting in the survival of cancer cells and accumulation of a large number of cells. In addition, An et al. (99) reported that BCL-2 not only inhibits apoptosis but also acts as an antagonist of tumor-suppressor genes. When these genes are mutated, lost, or inactivated, they can cause malignant transformation of cells and lead to the occurrence of tumors. For example, when BCL-2 is highly expressed in the body, cancer cells will resist drugs or chemotherapy during treatment, reducing the therapeutic effect of cancer. Indeed, BCL-2 is highly expressed *in vivo* when abnormal hyperplasia of gastric mucosa exists. The expression of BCL-2 was the highest in the early stage of gastric cancer and decreased gradually during the development of cancer.

### 3.2 BCL-2 and breast cancer

Breast cancer is mammary gland epithelial cells in a variety of carcinogenic factors below the action of proliferation out of control phenomenon which is a malignant tumor that seriously endangers women’s life and health worldwide. Its incidence is increasing year by year, and tends to be younger, accounting for about 25% of female malignant tumors (100). It is reported that breast cancer is a highly heterogeneous tumor, and its course of disease evolution has experienced complex biological processes such as multi-gene and multi-stage (101). Additionally, Merino et al. (102) found that the occurrence of breast cancer is not only related to the mutation, deletion, or activation of some proto-oncogenes and tumor-suppressor genes but also related to the inhibition of apoptosis. Actually, the BCL-2 gene involved in cell apoptosis is closely related to the occurrence of breast cancer and the proliferation of breast cancer cells. Raha et al. (103) reported that BCL-2 gene can not only inhibit apoptosis but also prolong the cell cycle and then delay tumor cell proliferation. Therefore, when the expression of BCL-2 is reduced, breast cancer may be induced. It was reported (104) that sick persons with high expression of BCL-2 gene had relatively good pathophysiological behavior, which can be used as one of the molecular biological indicators to predict the development of lymph node metastasis in cancer patients.

### 3.3 BCL-2 and lymphoma

Lymphoma is a malignant tumor originating from the lymphatic hematopoietic system and characterized by cell escape by apoptosis (105). During lymphoma formation, B cells are subjected to a wide range of stress stimuli (106), including oncogene activation, DNA damage (107), and oxygen and cytokine deficiency, all of which trigger apoptosis escape. Adams et al. (108) found the problem of high expression of BCL-W in B-cell lymphoma and proposed some clinical methods to inhibit anti-apoptotic BCL-2, making outstanding contributions to the exploration of how to treat B-cell lymphoma (109).

### 3.4 BCL-2 and prostate cancer

Prostate cancer, which occurs in the prostate epithelium, is the most common malignancy in men and is the second leading cause of cancer death in many European countries. There is growing interest in the molecular pathways of malignant transformation and progression of prostate cancer. Alterations in various oncogenes and tumor-suppressor genes can eventually lead to an imbalance between proliferation and programmed cell death, leading to net tumor growth and tumor progression (110). For instance, Bubendorf et al. (111) used immunohistochemical methods to detect the overexpression of BCL-2 in prostate cancer. Additionally, immunohistochemistry is particularly useful for BCL-2 analysis since there is always a strong positive in lymphocytes, basal cells, and peripheral nerve tissue, providing good internal control for each incision examined. Besides, overexpression of BCL-2 may promote the progression of prostate cancer by prolonging the net growth of tumors, thereby improving the survival rate of tumor cells.

### 3.5 BCL-2 and hepatocellular carcinoma

Hepatocellular carcinoma (HCC) is the second largest cancer-related death (112); the 5-year survival rate is only 18% (113), characterized by high mortality, strong invasiveness, low sensitivity to chemotherapy drugs, and easy resistance (114). Studies have shown that BCL-2 is highly expressed in HCC patients (115), and the BCL-2 family is contained in the mechanism of HCC chemoresistance (116). BCL-2 can prevent hepatocellular carcinoma cells from apoptosis and promote tumor formation mainly by blocking the Fas/FasL apoptosis pathway and forming a complex with BAX. BCL-2 inhibits apoptosis, and its overexpression and phosphorylation participate in the regulation of cell proliferation, playing an extremely important role in tumor formation and multidrug resistance. The study found (117) that mRNA and protein levels of BCL-2 were upregulated in HCC tissues. Chang et al. (118) found that the pcDNA3-F1 vector expressing FasL could significantly induce apoptosis of HCC cells, while the pcDNA3-FL-BCL-2 vector with high expression of BCL-2 could significantly block the apoptosis of HCC cells. Additionally, Wang et al. (119) reported gansu ammonia goose deoxycholic acid sodium glycochenodeoxycholate (GCDA) by enhancing BCL-2 in the family in T163 phosphorylation and promote resistant HCC cells.

### 3.6 BCL-2 and lung cancer

Lung cancer is one of the malignant tumors with the fastest-growing morbidity and mortality and the greatest threat to the health and life of the population (120). If the expression of BCL-2 is abnormal in lung cancer, the cells with irreparable genetic changes are prevented from dying and entering the cell cycle. The accumulation of various genetic changes can lead to tumorigenesis (121). For example, Meinhardt et al. (117) analyzed the role of BOK in lung cancer. By constructing a BOK−/− knockout mouse model, mice bearing the lox-stop-lox-KrasG12D allele were used and the mutant Kras was expressed in the lungs infected with AdenoCre virus. Subsequently, BOK-deficient mice reduced tumor burden by decreasing the number of lesions and histological grade and that BCL-2 family member BOK promoted Kras-driven lung cancer progression in a p53-dependent manner (122).

In addition, the overexpression of BCL-2 is an early event in the development of lung cancer. With the development of the disease, the growth rate and spread of lung cancer cells continue to increase, making the treatment very difficult. Emerging evidence reveals that early detection of BCL-2 expression level is of great significance for the treatment of patients with lung cancer. For example, Martin et al. (123) found that BCL-2 family members can be used as prognostic indicators for lung cancer, making outstanding contributions to further treatment and prevention of cancer. Moreover, Alam et al. (124) found that the EGFR pathway can modulate the role of the BAX/BCL-2 cascade in non-small-cell lung cancer (NSCLC). Inhibition of EGFR results in the upregulation of pro-apoptotic proteins that stimulate apoptosis by activating apoptotic pathways. These findings have important implications for the further treatment and prevention of lung cancer.

## 4 The mechanism of BCL-2 family proteins in regulating apoptosis

### 4.1 Relationship between apoptosis and necrosis, autophagy, and ROS generation

The most critical difference between apoptosis and necrosis is the integrity of the cell membrane (125). Apoptosis is the shrinkage of cells that maintains the integrity of the cell membrane and keeps the cell membrane wrapped even when the final cell fragment is formed. The biggest feature of this death method is that it can limit inflammation. The characteristic performance of cell necrosis is the destruction of the integrity of the cell membrane, the release of intracellular substances, and the release of intracellular substances can cause a significant inflammatory response (126). Among them, apoptotic necrosis and autophagy are both accompanied by mitochondrial permeability transition (127). Members of the BCL-2 family are also involved in these responses. P53 can activate or inhibit autophagy depending on cellular energy status and associated activation of other signaling pathways (128). Interactions between autophagy, apoptosis, and necrosis signaling jointly maintain T-cell homeostasis (129). Reactive oxygen species (ROS) are chemically reactive chemicals containing oxygen (130); ROS are formed as a natural by-product of the normal metabolism of oxygen and have important roles in cell signaling and homeostasis (131). ROS and mitochondria play pivotal roles in the induction of apoptosis under physiological and pathological conditions (132). Mitochondria are the main pro-apoptotic targets of excess reactive oxygen species (133), which can induce the opening of the permeable pores (PT pores) of the mitochondrial bilayer membrane and release calcium ions, cytochrome C, and apoptosis-inducing factor AIF, causing caspase caspase 9 to activate caspase 3/6/7. It can decouple the mitochondrial electron transport chain, downregulate the level of ATP production (134), upregulate the expression level of the pro-apoptotic protein BAX, and finally rupture the mitochondrial outer membrane, leading to apoptosis.

### 4.2 The mechanism of action of BCL-2 in regulating apoptosis

Three main pathways of apoptosis have been elucidated. Among them, one is the exogenous/death receptor pathway, which is activated by linking the death receptors in the TNF receptor superfamily (135) and contains three main receptor-inducing sub-apoptosis signal pathways, i.e., CD95/CD95L, TNFR, and AP03L/TRAILR pathways (136). The common feature of these pathways is that the initiation of the apoptosis signal is formed through the trimer of the receptor, and the initiation of caspases is recruited. Through stimulating the caspase cascade, apoptosis is caused (137). Additionally, the other apoptosis pathway is the mitochondria-independent pathway. Mitochondria, as the center of energy and metabolism in eukaryotes, also play a key role in regulating cell apoptosis signal transduction (138). The release of cytochrome C from mitochondria is a key step in apoptosis. In the presence of dATP, cytochrome C released into the cytoplasm can bind to APAF-1 to form a polymer and promote caspase-9 to bind to APAF-1 to form apoptotic bodies, and caspase-9 is activated (139). Activated Caspase-9 can induce other caspases such as caspase-3 and thus induce apoptosis. With respect the third apoptosis pathway, it is called the endoplasmic reticulum pathway recently discovered. The endoplasmic reticulum (ER) is a multipurpose organelle in cells, which is mainly responsible for maintaining the dynamic balance of cell functions (140). When the internal environment changes, it will cause the imbalance of ER homeostasis and thus induce endoplasmic reticulum stress (ERS). The early onset of ERS promotes cell survival, while long-term continuous ERS triggers the initiation of apoptosis pathways (141, 142).

Overexpression of BCL-2 can reduce oxygen free radical production and lipid peroxide formation. BCL-2 can reduce the transmembrane flow of calcium ions, suggesting that BCL-2 regulates apoptosis through calcium channels. Apoptotic factors accumulate on the endoplasmic reticulum and release Ca^2+^ (143), activating the precursor caspase-12, which in turn activates caspase-9 and caspase-3 and finally leads to apoptosis. The above three pathways finally converge to the same pathway; that is, activated caspase 8, caspase 9, and caspase 12 all cut and activate caspase 3, which ultimately leads to apoptosis.

For the first time, we found that the BCL-2 gene was translocated in chromosome (144) in follicular lymphoma T (14:18). Apoptosis plays a crucial role in tissue homeostasis, especially in the hematopoietic compartment, and its damage may cause tumor or autoimmune disease. Interactions between opposing components of the BCL-2 protein family largely determine whether stressed cells can survive. The BCL-2 family has four main functions: inhibitory effects (BCL-2, BCL-Xl, BCL-W, BCL2-A1, and MCL-1), activation (BIM and PUMA), effector (BAX and BAK), and sensitization (NOXA). BCL-2 and its closest homolog promote cell survival (145), but the other two factions promote apoptosis. In mammalian cells, they regulate the permeability of the outer mitochondrial membrane, most located on or transferred to the outer mitochondrial membrane. Only BH3-containing proteins can sense and transmit stress signals (146), but commitment to apoptosis requires either BAX or BAK. Only the proteins of BH3 appear to activate BAX and BAK indirectly by engaging and neutralizing their correlations, which would otherwise limit BAX and BAK by permeabilizing mitochondria. Additionally, the BCL-2 family autophagy and mitochondrial fission may also be subject to regulation of the BCL-2 family (147). Its pro-survival components are very attractive therapeutic targets for cancer, autoimmunity, and viral infections. Two hypotheses are now proposed about how its family regulates apoptotic apoptosis: direct and indirect models. The direct model indicates that BH3 proteins contain only BH3 protein components as stimulant or inhibitors: as stimulants, they directly activate BAX/BAK proteins to promote apoptosis, while the inhibitor BH3 protein activates BAX/BAK by releasing BIM, tBID, and PUMA by binding to anti-apoptotic members. The indirect model suggests that only the BH3-only protein binds to anti-apoptotic BCL-2 family members and then releases BAX/BAK to initiate apoptosis (148). There is evidence that these two mechanisms may coexist during the regulation of apoptosis.

## 5 Targeting BCL-2 family proteins for anticancer treatment

**Figure 3:**
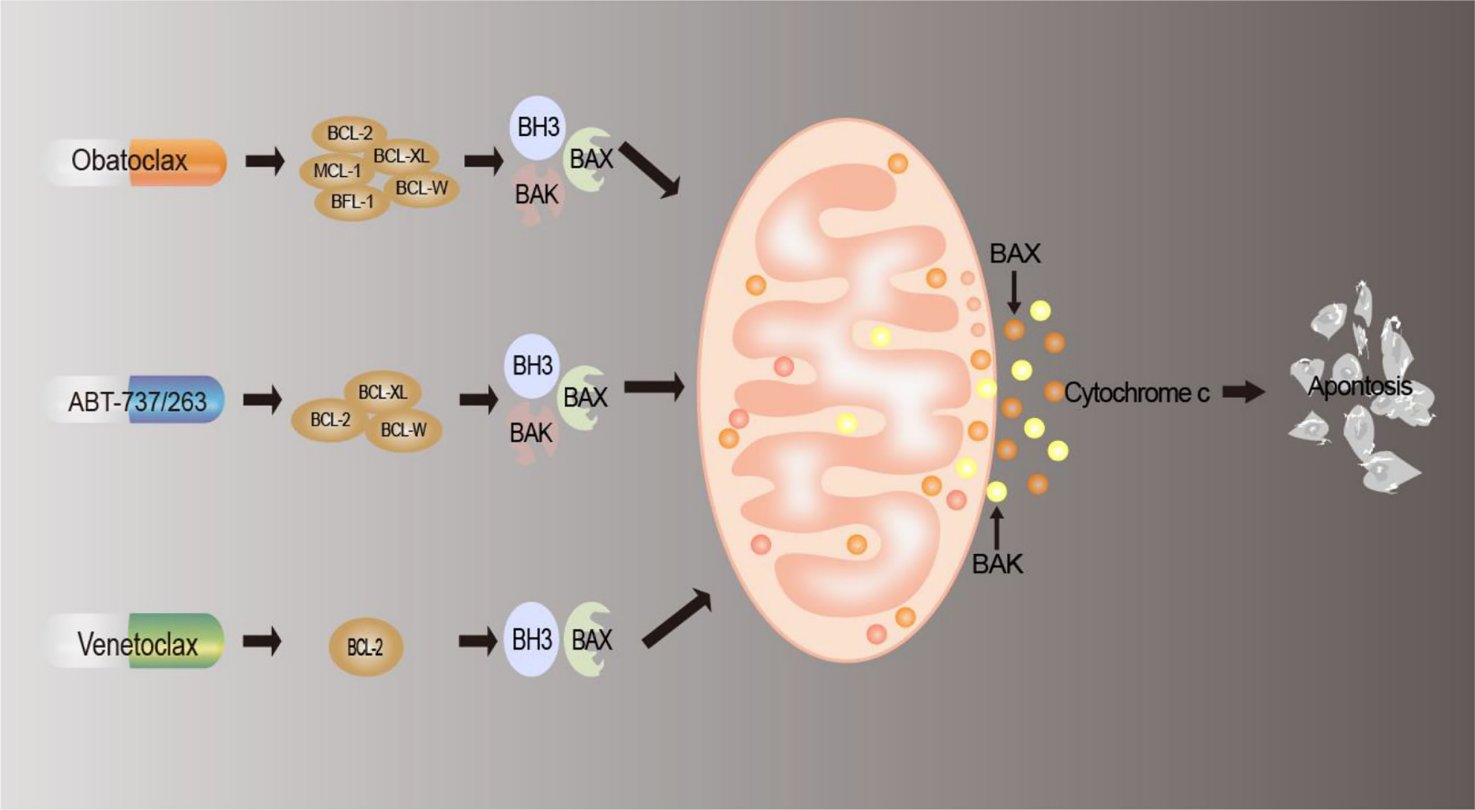
Mechanism of BCL-2 drug action. BCL-2 inhibitors interact with members of the BCL2 family of proteins to reduce the production of anti-apoptotic proteins, block the anti-apoptotic defense mechanism of tumor cells, replace and release pro-apoptotic proteins, induce apoptosis, and thus achieve antitumor effects.

The effective means of drug therapy for tumor cells currently include chemotherapy, targeted therapy, and immunotherapy, and apoptosis is the most important manifestation of cell death caused by these drugs (149). As we described above, deregulation of BCL-2 family proteins contributes to the development of cancer. Since the discovery of the BCL-2 family, people’s knowledge has changed from being involved in the regulation of cell survival to BCL-2 being a regulator of apoptosis (150), which is inseparable from tumor progression, tumor regression, and antagonism of cell death. At present, there are three types of inhibitors targeting the BCL-2 family at home and abroad: antisense oligonucleotide preparations (151), peptide inhibitors (152), and small-molecule inhibitors (153). Among them, small-molecule inhibitors are the most widely used and have the most research significance and development prospects (150). Therefore, the small-molecule inhibitors and their mechanisms of action are mainly described. The mechanism of action of small-molecule antagonists is shown in **Figure 3**, and a list of BCL-2 inhibitors is summarized in **Table 2**.

**Table 2:** Appellation, target, and clinical application of BCL-2 inhibitors.

Drug names	Target	Clinical application
Oblimersen (154)	mRNA of BCL-2	Myeloma (stage III)
Obatoclax (155)	BCL-2	CCL
	MCL-1	AML
	BCL-XL	NSCLC
	BFL-1	HL (stage II)
	BCL-W	SCLC (stage II)
Navitoclax (156)	BCL-2	Myeloma (stage I)
		CLL (stage I~III)
		NHL (stage I~III)
		AML (stage I~II)

### 5.1 Antisense oligonucleotide preparations

The principle behind the therapeutic strategy of antisense oligonucleotide preparations is to introduce a single-oligonucleotide strand complementary to the target sequence of the selected mRNA, forming a DNA heteroduplex, which is easily destroyed by RNase H, ultimately leading to a decrease in the level of the target mRNA (157).

### 5.2 Peptide inhibitors

Based on the pro-apoptotic program, BH3-only proteins exert their effects by directly binding or by binding anti-apoptotic family proteins to liberate BAX and BAK to activate both. Therefore, under such a research idea, a new type of hydrocarbon-labeled peptide, represented by the BIM-BH3 peptide, targeting the BIM-BH3 domain was born, which can effectively inhibit the interaction of BCL-2-BIM (152).

### 5.3 Small-molecule inhibitors

#### 5.3.1 BCL-2 inhibitors

**Figure 4:**
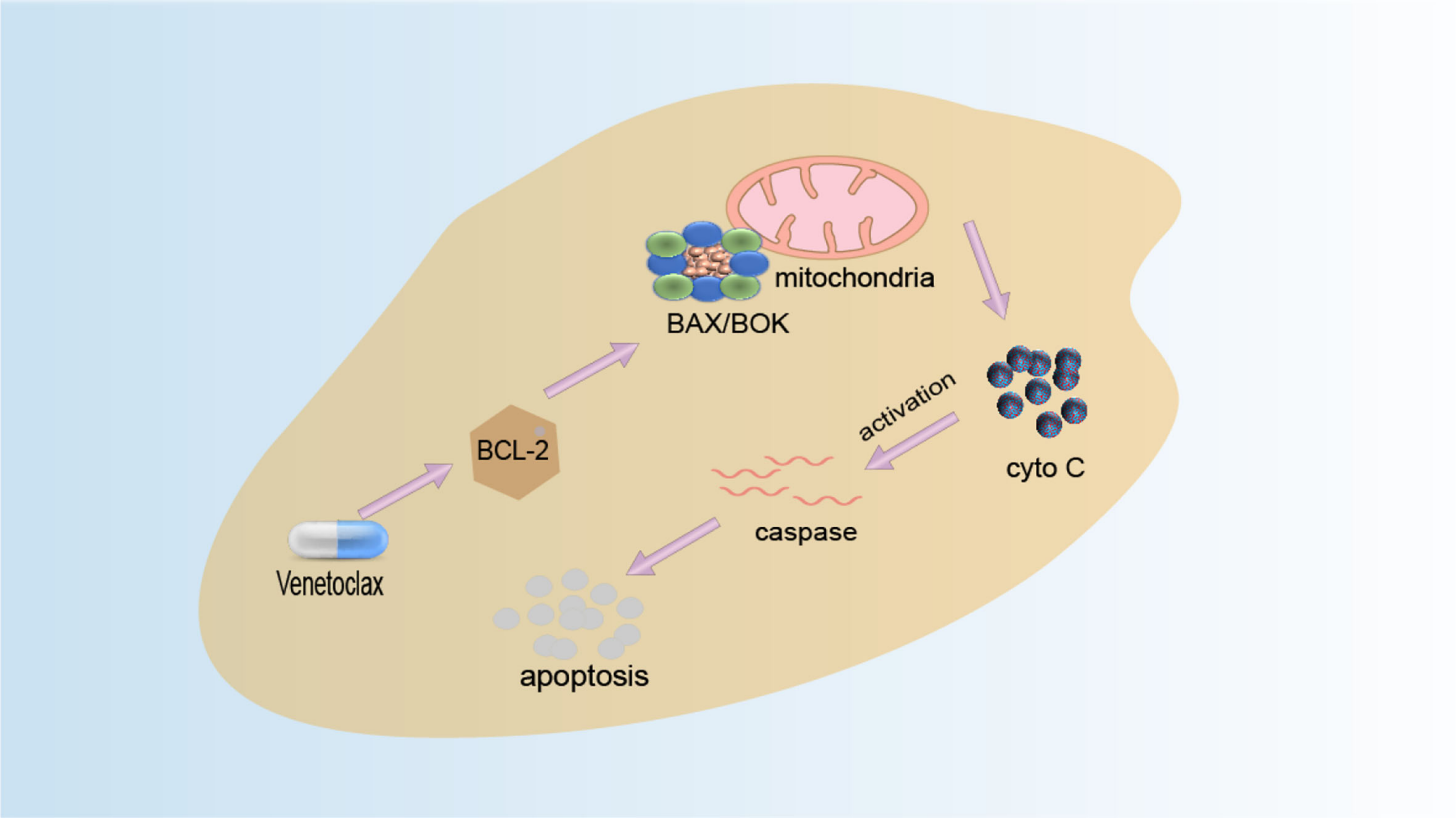
Venetoclax mechanism diagram. Venetoclax is a highly selective inhibitor of BCL-2. Highly expressed cancer cells of BCL-2 are spared from apoptosis by inhibiting the activation of BAX and BAK. Venetoclax selectively binds to BCL-2 in the binding tank, directly and indirectly reducing the inhibitory effect on BAX/BAK and initiating apoptosis.

The function of BCL-2 can be inhibited by BH3-only protein, and the binding site of BH3-only protein is also the binding site of BAX and BCL-2. Therefore, a reasonable anticancer drug design idea is to design BH3 analogs (158). Small-molecule compounds that mimic the BH3 domain readily enter cells and may selectively cause cell death with high expression of BCL-2 only. Venetoclax (VEN) is the first BCL-2 selective BH3 analogue which is an oral, potent, and selective BCL2 inhibitor and is currently the only anticancer drug of this type on the market. After the drug is absorbed into the human body, it acts on the BCL-2 protein (159). By selectively binding to BCL-2, it inhibits the production of anti-apoptotic protein BCL-2 and activates the interaction of pro-apoptotic proteins BAK, BAX, and mitochondria, thereby releasing cytochrome C, activating the apoptosis pathway, and causing apoptosis to achieve the effect of treating cancer. Its mechanism of action is shown in **Figure 4**. FDA approved it in 2016 for the treatment of patients with CLL and 17p deletion (160). Literature has found that patients have developed resistance to Venetoclax, for example, BCL-2 mutant G101V from clinical trials of phosphocytic leukemia patients who initially responded to treatment but whose clinical progression of CLL type emerged after 19–42 months. The BCL-2 G101V mutation reduced the drug’s affinity for BCL-2 by about 180-fold. On the other hand, BCL-2 G101V retains its affinity for the BH3 motif of pro-apoptotic proteins such as BAX and BIM and thus can still exert an anti-apoptotic effect. This suggests that the BCL-2G101V mutation confers resistance to treatment by selectively reducing affinity for venetoclax. Additionally, ABT-737 is the first BH3 analog designed by Abbott Laboratories based on high-throughput magnetic resonance imaging and the BAD structure of BH3-only protein and is a small molecule capable of targeted binding to the BH3 binding slot of BCL-XL (161). It can specifically bind to anti-apoptotic proteins to induce apoptosis. Moreover, obatoclax, a class of indole–pyrrole compounds, can bind to the BH3 domain of BCL-2 family proteins, thereby inhibiting the expression of BCL-2, BCL-XL, and MCL-1 proteins and inhibiting the expansion of tumor cells (162).

#### 5.3.2 BCL-2 antagonists

The loss or change of the hydrophobic structure leads to the inactivation of anti-apoptotic BCL-2 and the loss of its ability to bind to other members to form dimers (163). As a result, there are hundreds of homomorphic antagonists that mimic the BCL-2 BH3 domain. HA14-1, a small non-peptide organic ligand, interacts with the hydrophobic structure of BCL-2, which was also shown by computer screening and multiple cellular analyses to interact with soluble BCL-2, activating mitochondrial membrane potential changes and cytochrome C release and inducing apoptosis of tumor cells (164). At present, many studies have found (165–167) that the combination of HA14-1 with antitumor drugs (cisplatin) can enhance the pro-apoptotic effect of antitumor drugs.

## 6 Conclusions and future prospects

Accumulating evidence suggests that members of the BCL-2 family, as important regulators of apoptosis, play crucial roles in tumorigenesis, development, and treatment. Targeting the apoptotic pathway is an effective option to improve or develop new chemotherapy to help treat cancer, but it is necessary to systematically review the role of BCL-2 family proteins in regulating apoptosis and cancer treatment. Consequently, this review focused on the discussion of the role of BCL-2 family proteins in regulating the apoptosis and the development of drugs targeting BCL-2 anti-apoptotic proteins, although BCL-2 proteins have close correlations with apoptosis, necroptosis, autophagy, and ROS generation.

As demonstrated in the study, members of the BCL-2 family that inhibit apoptosis, such as BCL-2 or BCL-XL, are usually expressed in human tumor tissues at a high level, which inhibits the apoptosis of tumor cells and grows explosively. In view of this situation, if BCL-2 and BCL-XL can be functionally blocked, the apoptosis of tumor cells can be restored. In clinical applications, BCL-2 family molecules are very promising as tumor drug targets or biomarkers of tumor diseases, bringing hope to the targeted therapy of tumor diseases. Additionally, studies on the molecular mechanism of apoptosis have found that the BCL-2 family can be used for targeted therapy of tumors. At the same time, many members of this family can be used as tumor prognostic genes and have important effects on tumor prevention and treatment. Therefore, some efficient and specific antitumor drugs can be designed to treat malignant tumors by inhibiting the expression of anti-apoptotic proteins or activating the expression of pro-apoptotic proteins.

In addition, in the past few decades, a lot of research has been done to find BCL-2 inhibitors for cancer treatment, but there are still some prospective drugs that stay at the cellular level and rarely establish animal models. Continued research in this area should seek to define the cellular and molecular targets that control apoptosis and explore its potential for clinical translation. At the same time, of crucial importance in this field is the difference between anti-apoptotic and pro-apoptotic BCL-2 family proteins, and the effect of mutations on the function of this family of proteins.

## Author contributions

JW and YY designed the study. SQ, ZW, WY, JH wrote the manuscript and prepared figures. All authors contributed to the article and approved the submitted version.

## Acknowledgments

Thanks are given to the National Science Foundation for Young Scientists of China (Grant no. 81904062), the Natural Science Key Projects of Anhui University of Chinese Medicine (Grant no. 2020zrzd15 and no. 2021zrzd12), and the Natural Science Key Projects of Anshui universities (Grant no. KJ2020A0419, no. KJ2020A0394 and no. KJ2021A0577).

## Conflict of interest

The authors declare that the research was conducted in the absence of any commercial or financial relationships that could be construed as a potential conflict of interest.

## Publisher’s note

All claims expressed in this article are solely those of the authors and do not necessarily represent those of their affiliated organizations, or those of the publisher, the editors and the reviewers. Any product that may be evaluated in this article, or claim that may be made by its manufacturer, is not guaranteed or endorsed by the publisher.

## References

[1] Singh R Letai A Sarosiek K. Regulation of apoptosis in health and disease: the balancing act of BCL-2 family proteins. Nat Rev Mol Cell Biol (2019) 20(3):175–93. doi: 10.1038/s41580-018-0089-8 PMC7325303 30655609

[2] Nikoletopoulou V Markaki M Palikaras K Tavernarakis N. Crosstalk between apoptosis, necrosis and autophagy. Biochim Biophys Acta (BBA)-Molecular Cell Res (2013) 1833(12):3448–59. doi: 10.1016/j.bbamcr.2013.06.001 23770045

[3] Campbell KJ Tait SWG. Targeting BCL-2 regulated apoptosis in cancer. Open Biol (2018) 8(5):180002. doi: 10.1098/rsob.180002 29769323 PMC5990650

[4] Carneiro BA El-Deiry WS. Targeting apoptosis in cancer therapy. Nat Rev Clin Oncol (2020) 17(7):395–417. doi: 10.1038/s41571-020-0341-y 32203277 PMC8211386

[5] Luna-Vargas MPA Chipuk JE. The deadly landscape of pro-apoptotic BCL-2 proteins in the outer mitochondrial membrane. FEBS J (2016) 283(14):2676–89. doi: 10.1111/febs.13624 PMC4907887 26662859

[6] Buja LM. The cell theory and cellular pathology: Discovery, refinements and applications fundamental to advances in biology and medicine. Exp Mol Pathol (2021) 121:104660. doi: 10.1016/j.yexmp.2021.104660 34116021

[7] Valentin R Grabow S Davids MS. The rise of apoptosis: targeting apoptosis in hematologic malignancies. Blood (2018) 132(12):1248–64. doi: 10.1182/blood-2018-02-791350 30012635

[8] Bose P Gandhi V Konopleva M. Pathways and mechanisms of venetoclax resistance. Leukemia lymphoma (2017) 58(9):2026–39. doi: 10.1080/10428194.2017.1283032 PMC5478500 28140720

[9] Youle RJ Strasser A. The BCL-2 protein family: opposing activities that mediate cell death. Nat Rev Mol Cell Biol (2008) 9(1):47–59. doi: 10.1038/nrm2308 18097445

[10] Kang MH Reynolds CP. Bcl-2 inhibitors: targeting mitochondrial apoptotic pathways in cancer therapy. Clin Cancer Res (2009) 15(4):1126–32. doi: 10.1158/1078-0432.CCR-08-0144 PMC3182268 19228717

[11] Minn AJ Rudin CM Boise LH Thompson CB. Expression of bcl-xL can confer a multidrug resistance phenotype. Blood (1995) 86(5):1903–10. doi: 10.1182/blood.V86.5.1903.bloodjournal8651903 7655019

[12] Yoshino T Shiina H Urakami S Kikuno N Yoneda T Shigeno K. Bcl-2 expression as a predictive marker of hormone-refractory prostate cancer treated with taxane-based chemotherapy. Clin Cancer Res (2006) 12(20):6116–24. doi: 10.1158/1078-0432.CCR-06-0147 17062688

[13] Alexandrov LB Nik-Zainal S Wedge DC Aparicio SAJR Behjati S Biankin AV. Signatures of mutational processes in human cancer. Nature (2013) 500:415–21. doi: 10.1038/nature12477 PMC3776390 23945592

[14] Buljan M Blattmann P Aebersold R Boutros M. Systematic characterization of pan-cancer mutation clusters. Mol Syst Biol (2018) 14:e7974. doi: 10.15252/msb.20177974 29572294 PMC5866917

[15] Fresquet V Rieger M Carolis C García-Barchino MJ Martinez-Climent JA. Acquired mutations in BCL2 family proteins conferring resistance to the BH3 mimetic ABT-199 in lymphoma. Blood J Am Soc Hematol (2014) 123(26):4111–9. doi: 10.1182/blood-2014-03-560284 24786774

[16] Tahir SK Smith ML Hessler P Rapp LR Idler KB Park CH. Potential mechanisms of resistance to venetoclax and strategies to circumvent it. BMC Cancer (2017) 17(1):1–10. doi: 10.1186/s12885-017-3383-5 28578655 PMC5457565

[17] Blombery P Anderson MA Gong JN Thijssen R Birkinshaw RW Thompson ER. Acquisition of the recurrent Gly101Val mutation in BCL2 confers resistance to venetoclax in patients with progressive chronic lymphocytic LeukemiaBCL2 Gly101Val causes resistance to venetoclax in CLL. Cancer Discovery (2019) 9(3):342–53. doi: 10.1158/2159-8290.CD-18-1119 30514704

[18] Choi J Choi K Benveniste EN Rho SB Hong YS Lee JH. BCL-2 promotes invasion and lung metastasis by inducing matrix metalloproteinase-2. Cancer Res (2005) 65(13):5554–60. doi: 10.1158/0008-5472.CAN-04-4570 15994927

[19] Reed JC Zha H Aime-Sempe C Takayama S Wang HG. Structure–function analysis of bcl-2 family proteins. Mech lymphocyte activation Immune Regul VI (1996) 406:99–112. doi: 10.1007/978-1-4899-0274-0_10 8910675

[20] Petros AM Olejniczak ET Fesik SW. Structural biology of the bcl-2 family of proteins. Biochim Biophys Acta (BBA)-Molecular Cell Res (2004) 1644(2-3):83–94. doi: 10.1016/j.bbamcr.2003.08.012 14996493

[21] Gabellini C Trisciuoglio D Del Bufalo D. Non-canonical roles of bcl-2 and bcl-xL proteins: relevance of BH4 domain. Carcinogenesis (2017) 38(6):579–87. doi: 10.1093/carcin/bgx016 28203756

[22] Dadsena S Jenner A García-Sáez AJ. Mitochondrial outer membrane permeabilization at the single molecule level. Cell Mol Life Sci (2021) 78(8):3777–90. doi: 10.1007/s00018-021-03771-4 PMC8106609 33576840

[23] Dai H Meng W Kaufmann S. BCL2 family, mitochondrial apoptosis, and beyond. Cancer Trans Med (2016) 2(1):7–20. doi: 10.4103/2395-3977.177558

[24] Tilokani L Nagashima S Paupe V Prudent J.. Mitochondrial dynamics: overview of molecular mechanisms. Essays Biochem (2018) 62(3):341–60. doi: 10.1042/EBC20170104 PMC6056715 30030364

[25] Pinton P Ferrari D Magalhães P Schulze-Osthoff K Di Virgilio F Pozzan T. Reduced loading of intracellular Ca2+ stores and downregulation of capacitative Ca2+ influx in BCL-2–overexpressing cells. J Cell Biol (2000) 148(5):857–62. doi: 10.1083/jcb.148.5.857 PMC2174537 10704437

[26] Park HA Broman K Jonas EA. Oxidative stress battles neuronal bcl-xL in a fight to the death. Neural Regeneration Res (2021) 16(1):12. doi: 10.4103/1673-5374.286946 PMC7818872 32788441

[27] Arbab IA Looi CY Abdul AB Cheah FK Wong WF Sukari MA. Dentatin induces apoptosis in prostate cancer cells *via* BCL-2, bcl-xL, survivin downregulation, caspase-9,-3/7 activation, and NF-κB inhibition. Evidence-Based Complementary Altern Med (2012) 2012. doi: 10.1155/2012/856029 PMC3471446 23091559

[28] Siddiqui WA Ahad A Ahsan H. The mystery of BCL-2 family: BCL-2 proteins and apoptosis: an update. Arch Toxicol (2015) 89(3):289–317. doi: 10.1007/s00204-014-1448-7 25618543

[29] Lee EF Fairlie WD. The structural biology of bcl-xL. Int J Mol Sci (2019) 20(9):2234. doi: 10.3390/ijms20092234 PMC6540150 31067648

[30] González-García M Pérez-Ballestero R Ding L Duan L Boise LH Thompson CB. Bcl-XL is the major bcl-x mRNA form expressed during murine development and its product localizes to mitochondria. Development (1994) 120(10):3033–42. doi: 10.1242/dev.120.10.3033 7607090

[31] Bessou M Lopez J Gadet R Deygas M Popgeorgiev N Poncet D. The apoptosis inhibitor bcl-xL controls breast cancer cell migration through mitochondria-dependent reactive oxygen species production. Oncogene (2020) 39(15):3056–74. doi: 10.1038/s41388-020-1212-9 32066881

[32] Hartman ML Czyz M. BCL-w: apoptotic and non-apoptotic role in health and disease. Cell Death Dis (2020) 11(4):1–16. doi: 10.1038/s41419-020-2417-0 32317622 PMC7174325

[33] Hoppe T Matuschewski K Rape M Schlenker S Ulrich HD Jentsch S..Bcl-xl protein is beneficial to the survival of cardiac myocytes. Bcl-xl Protein prolonging survival time transplanted cardiac myocytes by reducing release mitochondrial cytochrome C prolonging Cold storage time cardiac myocytes (2000) 102(5):577–86. doi: 10.1016/s0092-8674(00)00080-5

[34] Gibson L Holmgreen SP Huang DC Bernard O Copeland NG Jenkins NA. Bcl-w,a novel member of the BCL-2 family, prom otes cell survival. Oncogene (1996) 13(4):665–75. 8761287

[35] Chauhan D Velankar M Brahmandam M Hideshima T Podar K Richardson P. A novel BCL-2/Bcl-XL/Bcl-w inhibitor ABT-737 as therapy in multiple myeloma. Oncogene (2007) 26(16):2374–80. doi: 10.1038/sj.onc.1210028 17016430

[36] Ayllón V Cayla X García A Fleischer A Rebollo A. The anti-apoptotic molecules bcl-xL and bcl-w target protein phosphatase 1α to bad. Eur J Immunol (2002) 32(7):1847–55. doi: 10.1002/1521-4141(200207)32:7<1847::AID-IMMU1847>3.0.CO;2-7 12115603

[37] O’Connor L Strasser A O’Reilly LA Hausmann G Adams JM Cory S. Bim: a novel member of the BCL-2 family that promotes apoptosis. EMBO J (1998) 17(2):384–95. doi: 10.1093/emboj/17.2.384 PMC1170389 9430630

[38] Zhu PJ Yu ZZ You QD Jiang ZY. Myeloid cell leukemin-1 inhibitors: A growing arsenal for cancer therapy. Drug Discov Today (2020) 25(10):1873–82. doi: 10.1016/j.drudis.2020.07.021 32771436

[39] O& apos Reilly LA Print C Hausmann G Moriishi K Cory S Huang DC. Tissue expression and subcellular localization of the pro-survival molecule bcl-w. Cell Death Differ (2001) 8(5):486–94. doi: 10.1038/sj.cdd.4400835 11423909

[40] Gong J Zhang JP Li B Zeng C You K Chen MX. MicroRNA-125b promotes apoptosis by regulating the expression of mcl-1, bcl-w and IL-6R. Oncogene (2013) 32(25):3071–9. doi: 10.1038/onc.2012.318 22824797

[41] Yamaguchi R Lartigue L Perkins G. Targeting mcl-1 and other BCL-2 family member proteins in cancer therapy. Pharmacol Ther (2019) 195:13–20. doi: 10.1016/j.pharmthera.2018.10.009 30347215

[42] Nazeri M Mirzaie-Asl A Saidijam M Moradi M. Methanolic extract of artemisia absinthium prompts apoptosis, enhancing expression of Bax/BCL-2 ratio, cell cycle arrest, caspase-3 activation and mitochondrial membrane potential destruction in human colorectal cancer HCT-116 cells. Mol Biol Rep (2020) 47(11):8831–40. doi: 10.1007/s11033-020-05933-2 33141288

[43] Harvey EP Hauseman ZJ Cohen DT Rettenmaier TJ Lee S Huhn AJ. Identification of a covalent molecular inhibitor of anti-apoptotic BFL-1 by disulfide tethering. Cell Chem Biol (2020) 27(6):647–56.e6. doi: 10.1016/j.chembiol.2020.04.004 32413285 PMC7405809

[44] Caro-Gómez LA Rosas-Trigueros JL Mixcoha E Vique-Sánchez JL Gasperin-Sánchez H Benítez-Cardoza CG. Exploring the conformational space of BCL-2 protein variants: dynamic contributions of the flexible loop domain and transmembrane region. Molecules (2019) 24(21):3896. doi: 10.3390/molecules24213896 PMC6865210 31671865

[45] Liu N Wang D Lian C Zhao R Tu L Zhang Y. Selective covalent targeting of anti-apoptotic BFL-1 by a sulfonium-tethered peptide. ChemBioChem (2021) 22(2):340–4. doi: 10.1002/cbic.202000473 32790056

[46] Kathania M Raje CI Raje M Dutta RK Majumdar S. Bflfl-1/A1 acts as a negative regulator of autophagy in mycobacteria infected macrophages. Int J Biochem Cell Biol (2011) 43:573e585. doi: 10.1016/j.biocel.2010.12.014 21167304

[47] Feng X Yan Z Zhou F Lou J Lyu X Ren X. Discovery of a selective and covalent small-molecule inhibitor of BFL-1 protein that induces robust apoptosis in cancer cells. Eur J Medicinal Chem (2022) 114327. doi: 10.1016/j.ejmech.2022.114327 35385805

[48] Cartron PF Gallenne T Bougras G Gautier F Manero F Vusio P. The first α helix of bax plays a necessary role in its ligand-induced activation by the BH3-only proteins bid and PUMA. Mol Cell (2004) 16(5):807–18. doi: 10.1016/j.molcel.2004.10.028 15574335

[49] Lalier L Cartron PF Juin P Nedelkina S Manon S Bechinger B. Bax activation and mitochondrial insertion during apoptosis. Apoptosis (2007) 12(5):887–96. doi: 10.1007/s10495-007-0749-1 17453158

[50] Hantusch A Das KK García-Sáez AJ Brunner T Rehm M. Bax retrotranslocation potentiates bcl-xL’s antiapoptotic activity and is essential for switch-like transitions between MOMP competency and resistance. Cell Death Dis (2018) 9(4):1–13. doi: 10.1038/s41419-018-0464-6 29567940 PMC5864878

[51] Edlich F. BCL-2 proteins and apoptosis: Recent insights and unknowns. Biochem Biophys Res Commun (2018) 500(1):26–34. doi: 10.1016/j.bbrc.2017.06.190 28676391

[52] Hauseman ZJ Harvey EP Newman CE Wales TE Bucci JC Mintseris J. Homogeneous oligomers of pro-apoptotic BAX reveal structural determinants of mitochondrial membrane permeabilization. Mol Cell (2020) 79(1):68–83.e7. doi: 10.1016/j.molcel.2020.05.029 32533918 PMC7472837

[53] Jeng PS Inoue-Yamauchi A Hsieh JJ Cheng EH. BH3-dependent and independent activation of BAX and BAK in mitochondrial apoptosis. Curr Opin Physiol (2018) 3:71–81. doi: 10.1016/j.cophys.2018.03.005 30334018 PMC6186458

[54] Lee HY Oh SH. Autophagy-mediated cytoplasmic accumulation of p53 leads to apoptosis through DRAM-BAX in cadmium-exposed human proximal tubular cells. Biochem Biophys Res Commun (2021) 534:128–33. doi: 10.1016/j.bbrc.2020.12.019 33321290

[55] Dai H Smith A Meng XW Schneider PA Pang YP Kaufmann SH. Transient binding of an activator BH3 domain to the bak BH3-binding groove initiates bak oligomerization. J Cell Biol (2011) 194(1):39–48. doi: 10.1083/jcb.201102027 21727192 PMC3135403

[56] Peña-Blanco A García-Sáez AJ. Bax, bak and beyond–mitochondrial performance in apoptosis. FEBS J (2018) 285(3):416–31. doi: 10.1111/febs.14186 28755482

[57] Chen PH Hsueh TC Wu JL Hong JR. Infectious spleen and kidney necrosis virus (ISKNV) triggers mitochondria-mediated dynamic interaction signals *via* an imbalance of Bax/Bak over BCL-2/Bcl-xL in fish cells. Viruses (2022) 14(5):922. doi: 10.3390/v14050922 35632664 PMC9144193

[58] Imre G. Cell death signalling in virus infection. Cell Signalling (2020) 76:109772. doi: 10.1016/j.cellsig.2020.109772 32931899 PMC7486881

[59] Luo X O’Neill KL Huang K. The third model of Bax/Bak activation: a BCL-2 family feud finally resolved? F1000Research (2020) 9. doi: 10.12688/f1000research.25607.1 PMC7411521 32802314

[60] Zhang H Holzgreve W De Geyter C. Evolutionarily conserved bok proteins in the BCL-2 family. FEBS Lett (2000) 480:311–3. doi: 10.1016/S0014-5793(00)01921-9 11034351

[61] Flores-Romero H Ros U Garcia-Saez AJ. Pore formation in regulated cell death. EMBO J (2020) 39(23):e105753. doi: 10.15252/embj.2020105753 33124082 PMC7705454

[62] Kondo S Shinomura Y Miyazaki Y Kiyohara T Tsutsui S Kitamura S. Mutations of the bak gene in human gastric and colorectal cancers. Cancer Res (2000) 60(16):4328–30. 10969770

[63] Jackson S Harwood C Thomas M Banks L Storey A. Role of bak in UV-induced apoptosis in skin cancer and abrogation by HPV E6 proteins. Genes Dev (2000) 14(23):3065–73. doi: 10.1101/gad.182100 PMC317098 11114894

[64] Westphal S Kalthoff H. Apoptosis: targets in pancreatic cancer. Mol Cancer (2003) 2(1):1–14. doi: 10.1186/1476-4598-2-6 12605713 PMC149420

[65] Carberry S D’Orsi B Monsefi N Salvucci M Bacon O Fay J. The BAX/BAK-like protein BOK is a prognostic marker in colorectal cancer. Cell Death Dis (2018) 9(2):1–10. doi: 10.1038/s41419-017-0140-2 29374142 PMC5833733

[66] Hsu SY Kaipia A A McGee,E Lomeli M Hsueh AJW. Bok is a pro-apoptotic BCL-2 protein with restricted expression in reproductive tissues and heterodimerizes with selective anti-apoptotic BCL-2 family members. Proc Natl Acad Sci USA (1997) 94:12401–6. doi: 10.1073/pnas.94.23.12401 PMC24966 9356461

[67] Schulman JJ Szczesniak LM Bunker EN Nelson HA Roe MW Ii LEW. Bok regulates mitochondrial fusion and morphology. Cell Death Differ (2019) 26:2682–94. doi: 10.1038/s41418-019-0327-4 PMC7224202 30976095

[68] Schulman J Szczesniak LM Bunker EN Nelson HA Roe MW Wagner LE. Bok regulates mitochondrial fusion and morphology. Cell Death Differentiation (2019) 26(12):2682–94. doi: 10.1038/s41418-019-0327-4 PMC7224202 30976095

[69] Shalaby R Flores-Romero H García-Sáez AJ. The mysteries around the BCL-2 family member BOK. Biomolecules (2020) 10(12):1638. doi: 10.3390/biom10121638 PMC7762061 33291826

[70] Roufayel R Younes K Al-Sabi A Murshid N. BH3-only proteins noxa and puma are key regulators of induced apoptosis. Life (2022) 12(2):256. doi: 10.3390/life12020256 35207544 PMC8875537

[71] Zhang Z Guo M Liu Y Liu P Cao X Xu Y. RNPS1 inhibition aggravates ischemic brain injury and promotes neuronal death. Biochem Biophys Res Commun (2020) 523(1):39–45. doi: 10.1016/j.bbrc.2019.11.185 31831174

[72] Fridman JS Parsels J Rehemtulla A Maybaum J. Cytochrome c depletion upon expression of bcl-XS. J Biol Chem (2001) 276(6):4205–10. doi: 10.1074/jbc.M008171200 11044452

[73] Lindenboim L Kringel S Braun T Borner C Stein R. Bak but not bax is essential for bcl-xS-induced apoptosis. Cell Death Differ (2005) 12(7):713–23. doi: 10.1038/sj.cdd.4401638 15861188

[74] Montero J Letai A. Why do BCL-2 inhibitors work and where should we use them in the clinic? Cell Death Differ (2018) 25(1):56–64. doi: 10.1038/cdd.2017.183 29077093 PMC5729538

[75] Liu J Liu W Yang H. Balancing apoptosis and autophagy for parkinson’s disease therapy: targeting BCL-2. ACS Chem Neurosci (2018) 10(2):792–802. doi: 10.1021/acschemneuro.8b00356 30400738

[76] Matthew-Onabanjo AN Janusis J Mercado-Matos J Carlisle AE Kim D Levine F. Beclin 1 promotes endosome recruitment of hepatocyte growth factor tyrosine kinase substrate to suppress tumor proliferation. Cancer Res (2020) 80(2):249–62. doi: 10.1158/0008-5472.CAN-19-1555 PMC6980665 31744816

[77] Luo Y Wu Y Huang H Yi N Chen Y. Emerging role of BAD and DAD1 as potential targets and biomarkers in cancer. Oncol Lett (2021) 22(6):1–13. doi: 10.3892/ol.2021.13072 PMC8503815 34671425

[78] Boac BM Abbasi F Ismail-Khan R Xiong Y Siddique A Park H. Expression of the BAD pathway is a marker of triple-negative status and poor outcome. Sci Rep (2019) 9(1):1–14. doi: 10.1038/s41598-019-53695-0 31767884 PMC6877530

[79] Wu W Yang B Qiao Y Zhou Q He H He M. Kaempferol protects mitochondria and alleviates damages against endotheliotoxicity induced by doxorubicin. Biomedicine Pharmacotherapy (2020) 126:110040. doi: 10.1016/j.biopha.2020.110040 32145585

[80] Westphal D Dewson G Czabotar PE Kluck RM. Molecular biology of bax and bak activation and action. Biochim Biophys Acta (2011) 1813(4):521–31. doi: 10.1016/j.bbamcr.2010.12.019 21195116

[81] O’Reilly LA Cullen L Visvader J Lindeman GJ Print C Bath ML. The proapoptotic BH3-only protein bim is expressed in hematopoietic, epithelial, neuronal, and germ cells. Am J Pathol (2000) 157(2):449–61. doi: 10.1016/S0002-9440(10)64557-9 PMC1850143 10934149

[82] Liu Q Osterlund EJ Chi X Pogmore J Leber B Andrews DW. Bim escapes displacement by BH3-mimetic anti-cancer drugs by double-bolt locking both bcl-XL and BCL-2. Elife (2019) 8:e37689. doi: 10.7554/eLife.37689 30860026 PMC6414199

[83] Mei AHC Tung K Han J Perumal D Laganà A Keats J. MAGE-a inhibit apoptosis and promote proliferation in multiple myeloma through regulation of BIM and p21Cip1. Oncotarget (2020) 11(7):727. doi: 10.18632/oncotarget.27488 32133047 PMC7041939

[84] Matuszyk J Klopotowska D. miR-125b lowers sensitivity to apoptosis following mitotic arrest: Implications for breast cancer therapy. J Cell Physiol (2020) 235(10):6335–44. doi: 10.1002/jcp.29610 32052874

[85] Lim Y De Bellis D Sandow JJ Capalbo L D'Avino PP Murphy JM. Phosphorylation by aurora b kinase regulates caspase-2 activity and function. Cell Death Differentiation (2021) 28(1):349–66. doi: 10.1038/s41418-020-00604-y PMC7852673 32811973

[86] Yin XM. Bid, a critical mediator for apoptosis induced by the activation of Fas/TNF-R1 death receptors in hepatocytes. J Mol Med (2000) 78(4):203–11. doi: 10.1007/s001090000099 10933582

[87] Ramachandran A Visschers RGJ Duan L Akakpo JY Jaeschke H. Mitochondrial dysfunction as a mechanism of drug-induced hepatotoxicity: current understanding and future perspectives. J Clin Trans Res (2018) 4(1):75. doi: 10.18053/jctres.04.201801.005 PMC6261533 30873497

[88] Greaves G Milani M Butterworth M Carter RJ Byrne DP Eyers PA. BH3-only proteins are dispensable for apoptosis induced by pharmacological inhibition of both MCL-1 and BCL-XL. Cell Death Differ (2019) 26(6):1037–47. doi: 10.1038/s41418-018-0183-7 PMC6748112 30185825

[89] Lee YS Lee DH Choudry HA Bartlett DL Lee YJ. Ferroptosis-induced endoplasmic reticulum stress: cross-talk between ferroptosis and apoptosis. Mol Cancer Res (2018) 16(7):1073–6. doi: 10.1158/1541-7786.MCR-18-0055 PMC6030493 29592897

[90] Hung CL Chang HH Lee SW Chiang YW. Stepwise activation of the pro-apoptotic protein bid at mitochondrial membranes. Cell Death Differ (2021) 28(6):1910–25. doi: 10.1038/s41418-020-00716-5 PMC8184993 33462413

[91] Nakano K Vousden KH. PUMA, a novel proapoptotic gene, is induced by p53. Mol Cell (2001) 7(3):683–94. doi: 10.1016/S1097-2765(01)00214-3 11463392

[92] Zheng C Liu T Liu H Wang J. Role of BCL-2 family proteins in apoptosis and its regulation by nutrients. Curr Protein Pept Sci (2020) 21(8):799–806. doi: 10.2174/1389203721666191227122252 31880257

[93] Sun YL Jiang WQ Luo QY Yang DJ Cai YC Huang HQ. A novel BCL-2 inhibitor, BM-1197, induces apoptosis in malignant lymphoma cells through the endogenous apoptotic pathway. BMC Cancer (2020) 20(1):1–12. doi: 10.1186/s12885-019-6169-0 PMC6938641 31892356

[94] Li M. The role of P53 up-regulated modulator of apoptosis (PUMA) in ovarian development, cardiovascular and neurodegenerative diseases. Apoptosis (2021) 26(5):235–47. doi: 10.1007/s10495-021-01667-z PMC8197724 33783663

[95] Li W He P Huang Y Li YF Lu J Li M. Selective autophagy of intracellular organelles: Recent research advances. Theranostics (2021) 11(1):222. doi: 10.7150/thno.49860 33391472 PMC7681076

[96] Saha A Saleem S Paidi RK Biswas SC. BH3-only proteins puma and Beclin1 regulate autophagic death in neurons in response to amyloid-β. Cell Death Discovery (2021) 7(1):1–13. doi: 10.1038/s41420-021-00748-x PMC8593071 34782612

[97] Han CW Lee HN Jeong MS Park SY Jang SB. Structural basis of the p53 DNA binding domain and PUMA complex. Biochem Biophys Res Commun (2021) 548:39–46. doi: 10.1016/j.bbrc.2021.02.049 33631672

[98] Adams JM Cory S. BCL-2-regulated apoptosis: mechanism and therapeutic potential. Curr Opin Immunol (2007) 19(5):488–96. doi: 10.1016/j.coi.2007.05.004 PMC2754308 17629468

[99] Ishida M Gomyo Y Tatebe S Ohfuji S Ito H. Apoptosis in humangastric mucosa, chronic gastritis, dysplasia, and carinoma: analysis by terminal deoxynucleotidyl transferase-mediated dUTP-biotin nick end labeling. Virchows Arch (1996) 428:229–35. doi: 10.1007/BF00196695 8764931

[100] Xu AG Li SG Liu JH Gan AH. Function of apoptosis and expression of the proteins BCL-2, p53 and c-myc in the development of gastric cancer. World J Gastroenterol (2001) 7(3):403–6. doi: 10.3748/wjg.v7.i3.403 PMC4688731 11819799

[101] Ilkhomovna KD. Morphological features of tumor in different treatment options for patients with locally advanced breast cancer. Int J Innovative Analyses Emerging Technol (2021) 1(2):4–5.

[102] Sun B Zhao H. The bioinformatics analysis of RIOX2 gene in lung adenocarcinoma and squamous cell carcinoma. PloS One (2021) 16(12):e0259447. doi: 10.1371/journal.pone.0259447 34855761 PMC8638848

[103] Merino D Lok Sw Visvader Je Lindeman GJ. Targeting BCL-2 to enhance vulnerability to therapy in estrogen receptor-positive breastc ancer. Oncogene (2016) 35(15):1877–7. doi: 10.1038/onc.2015.287 26257067

[104] Raha P Thomas S Thurn KT Park J Munster PN. Combined histone deacetylase inhibition and tamoxi fen induces apoptosis in tamoxifen-resistant breast cancer models, by reversing BCL-2 overexpr ession. Breast Cancer Res (2015) 17(1):: 26. doi: 10.1186/s13058-015-0533-z 25848915 PMC4367983

[105] Sirotković-Skerlev M Plavetić ND Sedlić F Kuna SK Vrbanec D Belev B. ‘ (2021) 30:95–104. doi: 10.3233/CBM-201497 PMC12499958 32986661

[106] de Charette M Houot R. Hide or defend, the two strategies of lymphoma immune evasion: potential implications for immunotherapy. Haematologica (2018) 103(8):1256. doi: 10.3324/haematol.2017.184192 30006449 PMC6068015

[107] Puccetti MV. The role of DNA replication fork remodeling proteins in lymphomagenesis and hematopoietic cell replication stress. (Doctoral dissertation) (2018).

[108] Knittel G Rehkämper T Nieper P. DNA Damage pathways and b-cell lymphomagenesis. Curr Opin Hematol (2018) 25(4):315–22. doi: 10.1097/MOH.0000000000000433 29702521

[109] Adams CM Clark-Garvey S Porcu P Eischen CM. Targeting the BCL-2 family in b cell lymphoma. Front Oncol (2019) 8:636. doi: 10.3389/fonc.2018.00636 30671383 PMC6331425

[110] Liu S Wang PP Chen CT Li D Liu QY Lv L. MicroRNA-148b enhances the radiosensitivity of b-cell lymphoma cells by targeting bcl-w to promote apoptosis. Int J Biol Sci (2020) 16(6):935. doi: 10.7150/ijbs.40756 32140063 PMC7053334

[111] Bubendorf L Sauter G Moch H Jordan P Blöchlinger A Gasser TC. Prognostic significance of BCL-2 in clinically localized prostate cancer. Am J Pathol (1996) 148(5):1557–65. PMC1861580 8623924

[112] Sia D Villanueva A Friedman SL Llovet JM. Liver cancer cell of origin, molecular class, and effects on patient prognosis. Gastroenterology (2017) 152(4):745. doi: 10.1053/j.gastro.2016.11.048 28043904 PMC12160040

[113] Craig AJ Von Felden J Garcia-Lezana T Sarcognato S Villanueva A. Tumour evolution in hepatocellular carcinoma. Nat Rev Gastroenterol Hep atol (2020) 17(3):139–52. doi: 10.1038/s41575-019-0229-4 31792430

[114] Lohitesh K Chowdhury R Mukherjee S. Resistance a major hindrance to chemotherapy in hepatocellular carcinoma: an insight. Cancer Cell Int (2018) 18(1):1–15. doi: 10.1186/s12935-018-0538-7 29568237 PMC5859782

[115] Maji S Panda S Samal SK Shriwas O Rath R Pellecchia M. BCL-2Antiapoptotic family proteins and chemoresistance in cancer. Adv Cancer Res (2018) 137:137:3. doi: 10.1016/bs.acr.2017.11.001 29405977

[116] Liu B Wang C Chen P Cheng B Cheng Y. RACKI induces chemotherapy resistance in esophageal carcinoma by upregulating the PI3K/AKT pathway and BCL-2 expression. OncoTargets Ther (2018) 11:211. doi: 10.2147/OTT.S152818 PMC5757499 29379302

[117] Liao Z Tan X Dong KS Zhang HW Chen XP Chu L. miRNA-448 inhibits cell growth by targeting BCL-2 in hepatocellular carcinoma. Digestive Liver Dis (2019) 51(5):703–11. doi: 10.1016/j.dld.2018.09.021 30316787

[118] Chang YC Xu YH. Expression of BCL-2 inhibited fas-mediated apoptosis in human hepatocellular carcinoma BEL7404 cells. Cell Res (2000) 10(3):233–42. doi: 10.1038/sj.cr.7290052 11032175

[119] Wang C Yang M Zhao J Li X Xiao X Zhang Y. Bile salt (glycochenodeoxycholate acid) induces cell survival and chemoresistance in hepatocellular carcinoma. J Cell Physiol (2019) 234(7):10899–906. doi: 10.1002/jcp.27905 30548625

[120] Chen S Wu S. Identifying lung cancer risk factors in the elderly using deep neural networks: quantitative analysis of web-based survey data. J Med Internet Res (2020) 22(3):e17695. doi: 10.2196/17695 32181751 PMC7109611

[121] Zhou X Ouyang S Li J Huang X Ai X Zeng Y. The novel non-immunological role and underlying mechanisms of B7-H3 in tumorigenesis. J Cell Physiol (2019) 234(12):21785–95. doi: 10.1002/jcp.28936 31222741

[122] Meinhardt AL Munkhbaatar E Höckendorf U Dietzen M Dechant M Anton M. The BCL-2 family member BOK promotes KRAS-driven lung cancer progression in a p53-dependent manner. Oncogene (2022) 41(9):1376–82. doi: 10.1038/s41388-021-02161-1 PMC8881215 35091677

[123] Martin B Paesmans M Berghmans T Branle F Ghisdal L Mascaux C. Role of BCL-2 as a prognostic factor for survival in lung cancer: a systematic review of the literature with meta-analysis. Br J Cancer (2003) 89(1):55–64. doi: 10.1038/sj.bjc.6601095 12838300 PMC2394216

[124] Alam M Alam S Shamsi A Adnan M Elasbali AM Al-Soud WA. Bax/BCL-2 cascade is regulated by the EGFR pathway: Therapeutic targeting of non-small cell lung cancer. Front Oncol (2022) 12:869672. doi: 10.3389/fonc.2022.869672 35402265 PMC8990771

[125] Hammerschmidt S Kuhn H Grasenack T Gessner C Wirtz H. Mechanisch induzierte apoptose und nekrose in alveolären typ-II-Zellen - beeinflussung durch captopril und l-arginin [Apoptosis and necrosis induced by cyclic mechanical stretching in alveolar type-II-cells–influence of captopril and l-arginine]. Pneumologie (2004) 58(4):222–9. doi: 10.1055/s-2004-818408 15098159

[126] Frangogiannis NG. Regulation of the inflammatory response in cardiac repair. Circ Res (2012) 110(1):159–73. doi: 10.1161/CIRCRESAHA.111.243162 PMC3690135 22223212

[127] Chaabane W User SD El-Gazzah M Jaksik R Sajjadi E Rzeszowska-Wolny J. Autophagy, apoptosis, mitoptosis and necrosis: interdependence between those pathways and effects on cancer. Arch Immunol Ther Exp (Warsz) (2013) 61(1):43–58. doi: 10.1007/s00005-012-0205-y 23229678

[128] Walsh CM Edinger AL. The complex interplay between autophagy, apoptosis, and necrotic signals promotes T-cell homeostasis. Immunol Rev (2010) 236:95–109. doi: 10.1111/j.1600-065X.2010.00919.x 20636811 PMC2966323

[129] Lemasters JJ Nieminen AL Qian T Trost LC Elmore SP Nishimura Y. The mitochondrial permeability transition in cell death: a common mechanism in necrosis, apoptosis and autophagy. Biochim Biophys Acta (1998) 1366(1-2):177–96. doi: 10.1016/s0005-2728(98)00112-1 9714796

[130] Bergamini CM Gambetti S Dondi A Cervellati C. Oxygen, reactive oxygen species and tissue damage. Curr Pharm Des (2004) 10(14):1611–26. doi: 10.2174/1381612043384664 15134560

[131] Lennicke C Cochemé HM. Redox metabolism: ROS as specific molecular regulators of cell signaling and function. Mol Cell (2021) 81(18):3691–707. doi: 10.1016/j.molcel.2021.08.018 34547234

[132] Simon HU Haj-Yehia A Levi-Schaffer F. Role of reactive oxygen species (ROS) in apoptosis induction. Apoptosis (2000) 5(5):415–8. doi: 10.1023/a:1009616228304 11256882

[133] Orrenius S. Reactive oxygen species in mitochondria-mediated cell death. Drug Metab Rev (2007) 39(2-3):443–55. doi: 10.1080/03602530701468516 17786631

[134] Yoshioka J Lee RT. Thioredoxin-interacting protein and myocardial mitochondrial function in ischemia–reperfusion injury. Trends Cardiovasc Med (2014) 24(2):75–80. doi: 10.1016/j.tcm.2013.06.007 23891554 PMC3870036

[135] Meng X Zhang J Wu H Yu D Fang X. Akkermansia muciniphila aspartic protease amuc_1434* inhibits human colorectal cancer LS174T cell viability *via* TRAIL-mediated apoptosis pathway. Int J Mol Sci (2020) 21(9):3385. doi: 10.3390/ijms21093385 PMC7246985 32403433

[136] Schneider P Tschopp J. Apoptosis induced by death receptors. Pharmacochemistry library (2000) 31:281–6. doi: 10.1016/S0165-7208(00)80030-6 10812970

[137] Guicciardi ME Gores GJ. Life and death by death receptors. FASEB J (2009) 23(6):1625–37. doi: 10.1096/fj.08-111005 PMC2698650 19141537

[138] Xia MF Zhang YZ Jin K Lu Z Zeng Z Xiong W. Communication between mitochondria and other organelles: a brand-new perspective on mitochondria in cancer. Cell Bioscience (2019) 9(1):1–19. doi: 10.1186/s13578-019-0289-8 30931098 PMC6425566

[139] Kalpage HA Bazylianska V Recanati MA Fite A Liu J Wan J. Tissue-specific regulation of cytochrome c by post-translational modifications: respiration, the mitochondrial membrane potential, ROS, and apoptosis. FASEB J (2019) 33(2):1540–53. doi: 10.1096/fj.201801417R PMC6338631 30222078

[140] Fernández A Ordóñez R Reiter RJ González-Gallego J Mauriz JL. Melatonin and endoplasmic reticulum stress: relation to autophagy and apoptosis. J Pineal Res (2015) 59(3):292–307. doi: 10.1111/jpi.12264 26201382

[141] Kim C Kim B. Anti-cancer natural products and their bioactive compounds inducing ER stress-mediated apoptosis: A review. Nutrients (2018) 10(8):1021. doi: 10.3390/nu10081021 PMC6115829 30081573

[142] Jeong SY Seol DW. The role of mitochondria in apoptosis. BMB Rep (2008) 41(1):11–22. doi: 10.5483/bmbrep.2008.41.1.011 18304445

[143] Kerr JFR Winterford CM Harmon BV. Apoptosis. Cancer (1993) 73:2013–20264. doi: 10.1002/1097-0142(19940415)73:8<2013::AID-CNCR2820730802>3.0.CO;2-J 8156506

[144] Tsujimoto Y Cossman J Jaffe E Croce CM. Involvement of the BCL-2 gene in human follicular lymphoma. Science (1985) 228(4706):1440–3. doi: 10.1126/science.3874430 3874430

[145] Suraweera CD Hinds MG Kvansakul M. Structural investigation of orf virus BCL-2 homolog orfv125 interactions with BH3-motifs from BH3-only proteins puma and hrk. Viruses (2021) 13(7):1374. doi: 10.3390/v13071374 34372579 PMC8310162

[146] Kelly PN Strasser A. The role of BCL-2 and its pro-survival relatives in tumourigenesis and cancer therapy. Cell Death Differ (2011) 18:1414e1424. doi: 10.1038/cdd.2011.17 21415859 PMC3149740

[147] Llambi F Moldoveanu T Tait SW Bouchier-Hayes L Temirov J McCormick LL. A unifified model of mammalian BCL-2 protein family interactions at the mitochondria, mol. Cell (2011) 44:517e531. doi: 10.1016/j.molcel.2011.10.001 PMC3221787 22036586

[148] Roberts JZ Crawford N Longley DB. The role of ubiquitination in apoptosis and necroptosis. Cell Death Differentiation (2021) 29:1–13. doi: 10.1038/s41418-021-00922-9 34912054 PMC8817035

[149] Vanneman M Dranoff G. Combining immunotherapy and targeted therapies in cancer treatment. Nat Rev Cancer (2012) 12(4):237–51. doi: 10.1038/nrc3237 PMC3967236 22437869

[150] Alam M Ali S Mohammad T Hasan GM Yadav DK Hassan MI. B cell lymphoma 2: a potential therapeutic target for cancer therapy. Int J Mol Sci (2021) 22(19):10442. doi: 10.3390/ijms221910442 34638779 PMC8509036

[151] Janaghard MS Erfani-Moghadam V Moghadam AAS. The BCL-2 silencing with an antisense oligonucleotide. Increase Early Apoptosis (2021) 1–16. doi: 10.21203/rs.3.rs-936588/v1

[152] Reddy CN Sankararamakrishnan R. Designing BH3-mimetic peptide inhibitors for the viral BCL-2 homologues A179L and BHRF1: Importance of long-range electrostatic interactions. ACS Omega (2021) 6(41):26976–89. doi: 10.1021/acsomega.1c03385 PMC8529603 34693118

[153] Wang S Yang D Lippman ME. Targeting BCL-2 and bcl-XL with nonpeptidic small-molecule antagonists[C]//Seminars in oncology. WB Saunders (2003) 30:133–42. doi: 10.1053/j.seminoncol.2003.08.015 14613034

[154] Touzeau C Maciag P Amiot M Moreau P. Targeting bcl-2 for the treatment of multiple myeloma. Leukemia (2018) 32(9):1899–907. doi: 10.1038/s41375-018-0223-9 30076373

[155] Mao B Le-Trilling VTK Wang K Mennerich D Hu J Zhao Z. Obatoclax inhibits SARS-CoV-2 entry by altered endosomal acidification and impaired cathepsin and furin activity *in vitro* . Emerging Microbes Infect (2022) 11(1):483–97. doi: 10.1080/22221751.2022.2026739 PMC8843317 34989664

[156] Kipps TJ Eradat H Grosicki S Catalano J Cosolo W Dyagil IS. A phase 2 study of the BH3 mimetic BCL2 inhibitor navitoclax (ABT-263) with or without rituximab, in previously untreated b-cell chronic lymphocytic leukemia. Leukemia Lymphoma (2015) 56(10):2826–33. doi: 10.3109/10428194.2015.1030638 PMC4643417 25797560

[157] Quemener AM Centomo ML Sax SL Panella R. Small drugs, huge impact: The extraordinary impact of antisense oligonucleotides in research and drug development. Molecules (2022) 27(2):536. doi: 10.3390/molecules27020536 35056851 PMC8781596

[158] Rasmussen ML Gama V. A connection in life and death: The BCL-2 family coordinates mitochondrial network dynamics and stem cell fate. Int Rev Cell Mol Biol (2020) 353:255–84. doi: 10.1016/bs.ircmb.2019.12.005 PMC7331972 32381177

[159] Juárez-Salcedo LM Desai V Dalia S. Venetoclax: evidence to date and clinical potential. Drugs Context (2019) 8:8. doi: 10.7573/dic.212574 PMC6788387 31645879

[160] Opydo-Chanek M Gonzalo O Marzo I. Multifaceted anticancer activity of BH3 mimetics: Current evidence and future prospects. Biochem Pharmacol (2017) 136:12–23. doi: 10.1016/j.bcp.2017.03.006 28288819

[161] Opydo-Chanek M Cichoń I Rak A Kołaczkowska E Mazur L. The pan-BCL-2 inhibitor obatoclax promotes differentiation and apoptosis of acute myeloid leukemia cells. Invest N Drugs (2020) 38(6):1664–76. doi: 10.1007/s10637-020-00931-4 PMC7575496 32367199

[162] Souers AJ Leverson JD Boghaert ER Ackler SL Catron ND Chen J. ABT-199, a potent and selective BCL-2 inhibitor, achieves antitumor activity while sparing platelets. Nat Med (2013) 19(2):202–8. doi: 10.1038/nm.3048 23291630

[163] Yang S Mao Y Zhang H Xu Y An J Huang Z. The chemical biology of apoptosis: revisited after 17 years. Eur J Medicinal Chem (2019) 177:63–75. doi: 10.1016/j.ejmech.2019.05.019 31129454

[164] Verdine GL Walensky LD. The challenge of drugging undruggable targets in cancer: lessons learned from targeting BCL-2 family members. Clin Cancer Res (2007) 13(24):7264–70. doi: 10.1158/1078-0432.CCR-07-2184 18094406

[165] Varela FA Foust VL Hyland TE Sala-Hamrick KE Mackinder JR Martin CE. TMPRSS13 promotes cell survival, invasion, and resistance to drug-induced apoptosis in colorectal cancer. Sci Rep (2020) 10(1):1–14. doi: 10.1038/s41598-020-70636-4 32807808 PMC7431588

[166] Arisan ED Kutuk O Tezil T Bodur C Telci D Basaga H. Small inhibitor of BCL-2, HA14-1, selectively enhanced the apoptotic effect of cisplatin by modulating BCL-2 family members in MDA-MB-231 breast cancer cells. Breast Cancer Res Treat (2010) 119(2):271–81. doi: 10.1007/s10549-009-0343-z 19238538

[167] Simonin K Brotin E Dufort S Dutoit S Goux D N'diaye M. Mcl-1 is an important determinant of the apoptotic response to the BH3-mimetic molecule HA14-1 in cisplatin-resistant ovarian carcinoma cells. Mol Cancer Ther (2009) 8(11):3162–70. doi: 10.1158/1535-7163.MCT-09-0493 19887550

